# Reduction of exacerbations by the PDE4 inhibitor roflumilast - the importance of defining different subsets of patients with COPD

**DOI:** 10.1186/1465-9921-12-18

**Published:** 2011-01-27

**Authors:** Stephen I Rennard, Peter MA Calverley, Udo M Goehring, Dirk Bredenbröker, Fernando J Martinez

**Affiliations:** 1 Nebraska Medical Center, Omaha, USA; 2 University Hospital Aintree, Liverpool, UK; 3 Nycomed GmbH, Konstanz, Germany; 4 University of Michigan Medical Center, Ann Arbor, USA

## Abstract

**Background:**

As chronic obstructive pulmonary disease (COPD) is a heterogeneous disease it is unlikely that all patients will benefit equally from a given therapy. Roflumilast, an oral, once-daily phosphodiesterase 4 inhibitor, has been shown to improve lung function in moderate and severe COPD but its effect on exacerbations in unselected populations was inconclusive. This led to the question of whether a responsive subset existed that could be investigated further.

**Methods:**

The datasets of two previous replicate, randomized, double-blind, placebo-controlled, parallel-group studies (oral roflumilast 500 μg or placebo once daily for 52 weeks) that were inconclusive regarding exacerbations were combined in a post-hoc, pooled analysis to determine whether roflumilast reduced exacerbations in a more precisely defined patient subset.

**Results:**

The pooled analysis included 2686 randomized patients. Roflumilast significantly decreased exacerbations by 14.3% compared with placebo (p = 0.026). Features associated with this reduction were: presence of chronic bronchitis with or without emphysema (26.2% decrease, p = 0.001), presence of cough (20.9% decrease, p = 0.006), presence of sputum (17.8% decrease, p = 0.03), and concurrent use of inhaled corticosteroids (ICS; 18.8% decrease, p = 0.014). The incidence of adverse events was similar with roflumilast and placebo (81.5% vs 80.1%), but more patients in the roflumilast group had events assessed as likely or definitely related to the study drug (21.5% vs 8.3%).

**Conclusions:**

This post-hoc, pooled analysis showed that roflumilast reduced exacerbation frequency in a subset of COPD patients whose characteristics included chronic bronchitis with/without concurrent ICS. These observations aided the design of subsequent phase 3 studies that prospectively confirmed the reduction in exacerbations with roflumilast treatment.

**Trials registration:**

ClinicalTrials.gov identifiers: NCT00076089 and NCT00430729.

## Background

Chronic obstructive pulmonary disease (COPD) is a highly prevalent condition and a major cause of morbidity and mortality worldwide [1-3]. As the disease progresses, patients with COPD report more frequent exacerbations, which are associated with an increased mortality risk and greater health care utilization, hospital admissions and costs [4]. Worse, frequent exacerbations are associated with a faster decline in lung function and increased mortality [5].

Phosphodiesterase 4 (PDE4) inhibitors are effective anti-inflammatory agents in animal models and have been shown to reduce markers of inflammation in COPD [6,7]. In a 6-month study in patients with moderate-to-severe COPD (post-bronchodilator mean forced expiratory volume in 1 second [FEV_1_] 54% predicted [8]), the PDE4 inhibitor roflumilast improved lung function and reduced exacerbations [9]. This led to two subsequent 12-month studies (M2-111, reported here for the first time, and M2-112 [10]) in patients with severe-to-very-severe COPD, which confirmed the positive effect of roflumilast on lung function. Although neither study demonstrated a significant effect on exacerbations, which was a co-primary endpoint, a trend towards lower overall exacerbation rates with roflumilast was seen in each study.

As COPD is a highly heterogeneous disease [11], the possibility that a subset of the COPD population might be more responsive to roflumilast-induced reduction in exacerbations was entertained. To test this hypothesis, the results from the two 12-month studies, that were inconclusive with regard to exacerbations, were pooled and a series of post-hoc analyses performed. The results of these analyses are presented in the current report. The heterogeneity of the COPD patient population is well recognized. However, clinically meaningful subsets of patients with COPD have been difficult to define and several large observational studies are currently underway to attempt to address this problem [12-14]. The current post-hoc analysis of pooled clinical trial data was conducted in order to define a subset of patients with COPD who are likely to respond to a specific therapy - a 'hypothesis-generating' exercise that has been confirmed in subsequent clinical trials [15]. The approach described in the current study may be applicable to define other meaningful subsets of patients with COPD.

## Methods

### Patients and study design

M2-111 was conducted between December 2003 and December 2005 in 188 centers in 6 countries, and M2-112 between January 2003 and October 2004 in 159 centers in 14 countries. Full details of the methodology, patient selection and efficacy assessments have been published previously for M2-112 [10]. (For details of the clinical design of both trials, and a CONSORT diagram for the unpublished study M2-111, see Additional file 1, Appendix 1, and Additional file 1, Figure S1).

The studies were approved by local ethical review committees (see Additional file 1, Appendix 2 for a list of committee names and approval numbers) and performed in accordance with the Declaration of Helsinki and Good Clinical Practice Guidelines.

### Statistical analysis

The statistical analysis was performed as described previously [10] with some modifications (i.e., all data were re-analyzed based on the methods used in two other 52-week studies) [15]. The primary endpoint (pre-bronchodilator FEV_1_) and main secondary lung function endpoint (post-bronchodilator FEV_1_) were evaluated using a repeated measures analysis of covariance (ANCOVA, mixed effects model). This model is able to handle missing data points by taking into account all available data from scheduled visits of the treatment period and the correlation in repeated measurements. The co-primary endpoint of rate of moderate or severe exacerbations per patient per year was defined by the need for oral or parenteral corticosteroid treatment, hospitalization, or death, and was evaluated using a Poisson regression model with a correction for over-dispersion. The natural logarithm of the trial duration, in terms of years, was included in this model as an offset variable to correct for the time a patient participated in the trial. Rate ratios from this model were expressed as percent reductions. Time to onset of exacerbations was analyzed using a Cox proportional hazards regression model. For the regression models (ANCOVA, Poisson, and Cox), the covariates included treatment (roflumilast/placebo), age, sex, smoking status (current/former smoker), study, concomitant treatment with inhaled corticosteroids (ICS) and country pool (only for the overall population). In the Poisson regression analysis, baseline post-bronchodilator FEV_1 _(% of predicted value) was also included as a covariate. Adverse events were analyzed using descriptive statistics.

Data are presented as mean and standard deviation (SD), unless otherwise indicated. Safety endpoints were analyzed using descriptive statistics. Results are presented as mean ± SD or standard error (SE) as appropriate, with data derived from the statistical modeling being adjusted means. All p values are reported two-sided with a level of significance of 0.05.

To identify subpopulations, the two primary endpoints were analyzed additionally in subgroups stratified by sex, smoking status, concomitant use of ICS, concomitant use of anticholinergics, study completion status, COPD severity (severe, very severe), history of chronic bronchitis or emphysema (investigator-diagnosed), as well as cough and sputum score during the week before randomization.

## Results

### Patients

**Figure 1:**
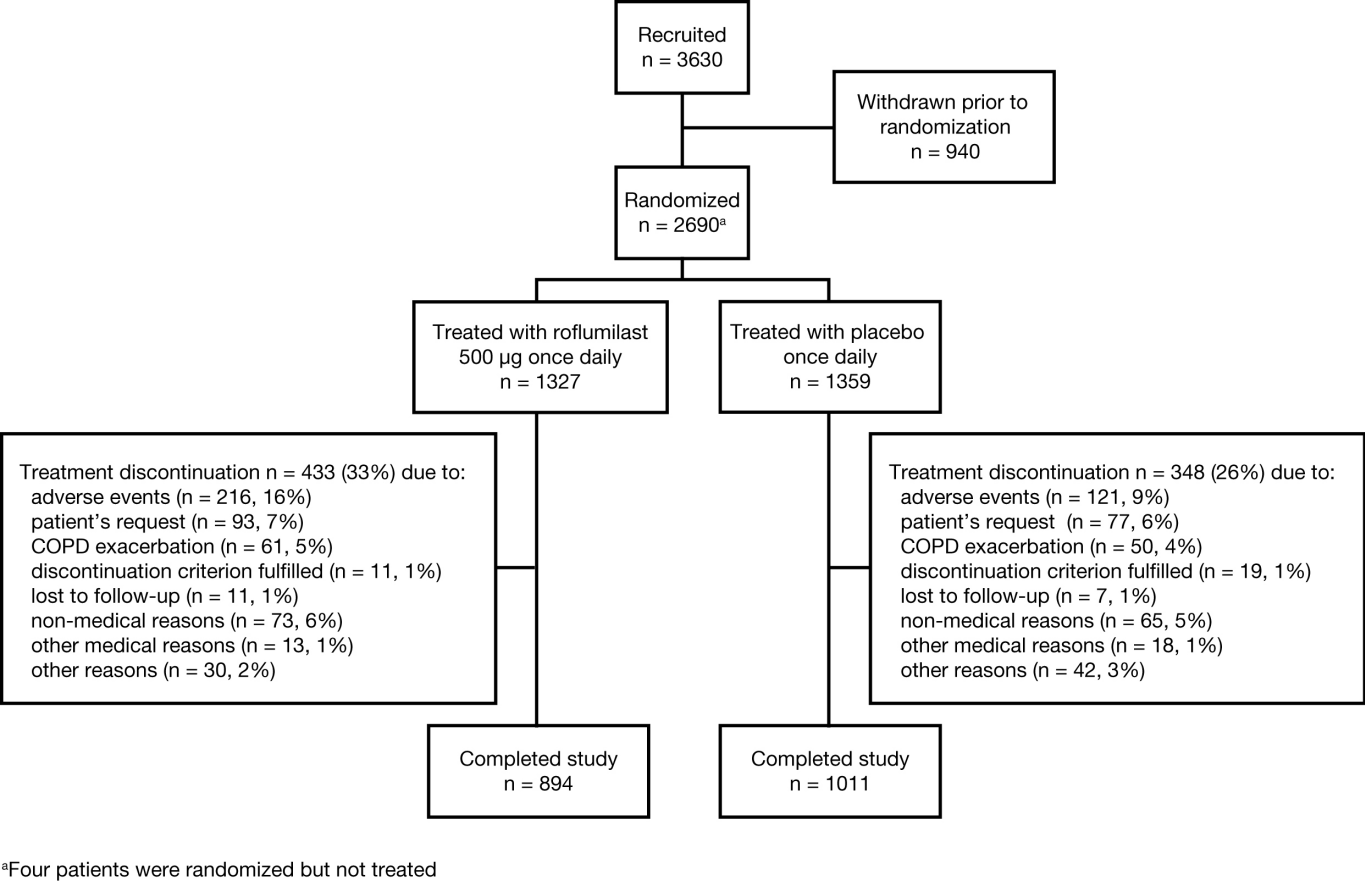
**Trial profiles of M2-111 and M2-112**. Percentages are based on the number of randomized patients in a treatment group.

Of 3630 patients enrolled into the run-in period, 2686 patients met the inclusion criteria and were randomized to treatment; 1905 patients completed the studies (Figure 1). The reasons for withdrawal were similar between groups except for adverse events, which occurred more frequently with roflumilast.

Demographics and baseline characteristics of the randomized patients were comparable between treatments (Table 1). Patients were predominantly male, and spirometric severity was consistent with severe-to-very-severe disease [8]. FEV_1 _reversibility to short-acting β_2_-agonists was similar in both treatment groups. As the inclusion criterion of FEV_1 _reversibility to short-acting β_2_-agonists ≤15% was defined only in study M2-112, mean reversibility was lower in M2-112 (11%) than in M2-111 (19%). All other demographic and baseline characteristics were comparable (or with only small differences not considered clinically relevant) between the two studies. On study entry and during the course of the studies, about 60% of the patients continued to receive ICS, while 60% continued to receive short-acting anticholinergics (Table 1).

**Table 1:** Demographics and baseline characteristics

	Pooled study population		M2-111		M2-112	
	
Characteristics	Roflumilast	Placebo	Roflumilast	Placebo	Roflumilast	Placebo
No. of patients	1327	1359	567	606	760	753

Age (years)	64.7 (9.2)	64.4 (8.9)	64 (8.7)	64 (8.8)	65 (9.6)	64 (9.1)

Male sex, n (%)	958 (72.2)	974 (71.7)	387 (68.3)	400 (66.0)	571 (75.1)	574 (76.2)

Body mass index, kg/m^2^	25.7 (5.3)	25.7 (5.4)	26.0 (5.7)	25.8 (5.7)	25.4 (5.0)	25.6 (5.1)

Smoking status						
Current smokers, n (%)	529 (40)	530 (39)	240 (42)	265 (44)	289 (38)	265 (35)
Former smokers, n (%)	798 (60)	829 (61)	327 (58)	341 (56)	471 (62)	488 (65)
Pack-years (± SD)	46 (25.6)	48 (26.6)	50 (28.2)	51 (26.7)	42 (22.9)	45 (26.2)

Pre-bronchodilator FEV_1 _(L)	1.0 (0.4)	1.0 (0.3)	0.96 (0.4)	0.93 (0.3)	1.04 (0.4)	1.06 (0.3)

Post-bronchodilator FEV_1 _(L)	1.13 (0.4)	1.13 (0.4)	1.12 (0.4)	1.09 (0.4)	1.13 (0.4)	1.15 (0.4)

Post-bronchodilator FEV_1 _(% predicted)	37.1 (10.5)	36.8 (9.9)	36.8 (10.7)	36.1 (9.7)	37.3 (10.3)	37.3 (9.9)

Reversibility:						
Change in FEV_1 _(mL)	126.9 (140.1)	125.8 (149.1)	165.6 (142.8)	160.9 (150.0)	98.1 (130.9)	97.6 (142.4)
Change in FEV_1 _(%)	14.6 (16.4)	14.4 (16.4)	19.4 (17.1)	19.1 (17.6)	11.0 (14.8)	10.6 (14.4)

FEV_1_/FVC (%)	41.8 (11.3)	41.8 (10.7)	43.3 (10.7)	43.1 (10.1)	40.6 (11.5)	40.7 (11.2)

COPD severity, n (%)						
Very severe COPD	329 (24.8)	345 (25.4)	148 (26.1)	169 (27.9)	181 (23.8)	176 (23.4)
Severe COPD	864 (65.1)	909 (66.9)	356 (62.8)	399 (65.8)	508 (66.8)	510 (67.7)

COPD history, n (%)						
Emphysema	352 (26.5)	413 (30.4)	193 (34.0)	234 (38.6)	159 (20.9)	179 (23.8)
Chronic bronchitis ± emphysema	817 (61.6)	847 (62.3)	374 (66.0)	372 (61.4)	443 (58.3)	475 (63.1)

Pre-study medication for COPD, n (%)*	1273 (96)	1291 (95)	537 (95)	557 (92)	736 (97)	734 (98)
Inhaled short-acting β agonists	729 (55)	734 (54)	315 (56)	333 (55)	414 (55)	401 (53)
Inhaled corticosteroids	579 (44)	588 (43)	218 (38)	225 (37)	361 (48)	363 (48)
Inhaled short-acting anticholinergics	549 (41)	570 (42)	189 (33)	192 (32)	360 (47)	378 (50)
Inhaled long-acting β_2_-agonists	353 (27)	379 (28)	143 (25)	140 (23)	210 (28)	239 (32)
Xanthines	320 (24)	316 (23)	113 (20)	118 (20)	207 (27)	198 (26)
Inhaled combination of β_2_-agonists and short-acting anticholinergics	323 (24)	314 (23)	168 (30)	174 (29)	155 (20)	140 (19)
Inhaled combination of corticosteroids and long-acting β_2_-agonists	260 (20)	263 (19)	131 (23)	139 (23)	129 (17)	124 (17)

Concomitant short-acting anticholinergics, n (%)	786 (59)	818 (60)	334 (59)	350 (58)	452 (60)	468 (62)

Concomitant inhaled corticosteroids, n (%)	809 (61)	813 (60)	328 (58)	332 (55)	481 (63)	481 (64)

Data are expressed as mean (SD), unless otherwise stated.

* Patients could have received more than one of these medications.

### Exacerbations

**Figure 2:**
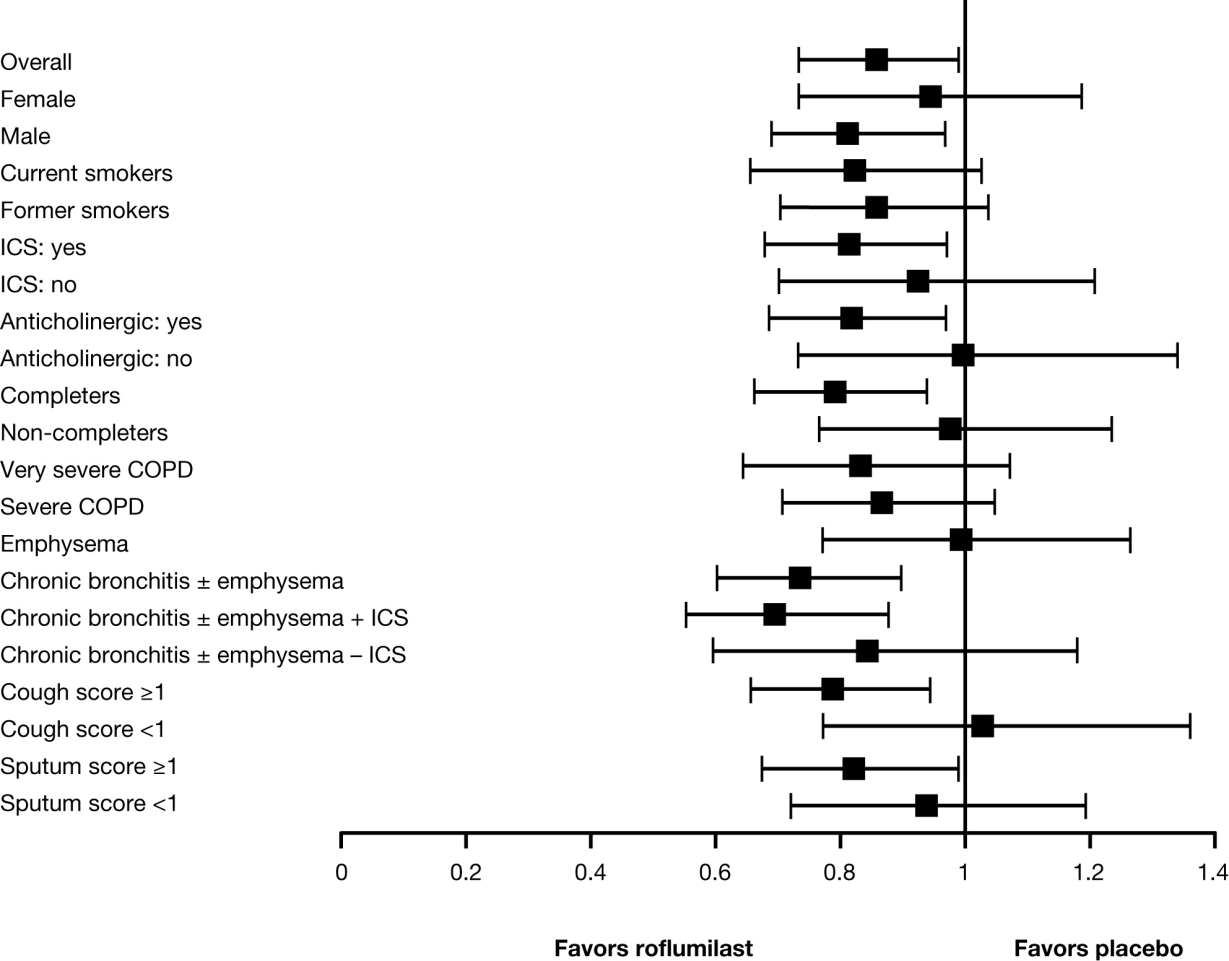
**Rate ratios and 95% CIs for reduction in COPD exacerbations with roflumilast by patient subgroup**. Error bars represent 95% CIs.

The rate of moderate-to-severe exacerbations in the pooled analysis was 14.3% lower with roflumilast compared with placebo (0.52 vs 0.61 exacerbations per year; p = 0.026, Table 2 and Figure 2). However, the median time to first moderate or severe exacerbation was comparable in the roflumilast and placebo groups (120 and 126 days, respectively, p = 0.236).

**Table 2:** Analysis of exacerbations (moderate to severe)

	Roflumilast		Placebo		Effect size		
	
Characteristic	n	Rate	n	Rate	Rate ratio (SE)	Change (%)	p value
M2-111	567	0.595	606	0.692	0.860 (0.085)	-14.0	0.129
M2-112	760	0.455	753	0.537	0.848 (0.081)	-15.2	0.085

Pooled results							

Overall	1327	0.523	1359	0.610	0.857 (0.059)	-14.3	0.026

Sex							
Female	369	0.612	385	0.648	0.943 (0.117)	-5.7	0.637
Male	958	0.495	974	0.609	0.813 (0.071)	-18.7	0.018

Smoking status							
Current smoker	529	0.529	530	0.643	0.823 (0.094)	-17.7	0.086
Former smoker	798	0.568	829	0.663	0.857 (0.078)	-14.3	0.092

Concomitant treatment							
ICS	809	0.720	813	0.886	0.812 (0.068)	-18.8	0.014
No ICS	518	0.424	546	0.460	0.923 (0.124)	-7.7	0.550

Concomitant treatment							
Short-acting anticholinergics	786	0.706	818	0.864	0.817 (0.066)	-18.3	0.012
No short-acting anticholinergics	541	0.368	541	0.370	0.995 (0.147)	-0.5	0.974

COPD severity							
Very severe COPD	329	0.738	345	0.885	0.833 (0.101)	-16.7	0.132
Severe COPD	864	0.526	909	0.609	0.864 (0.080)	-13.6	0.113

COPD history							
Emphysema	352	0.579	413	0.586	0.989 (0.120)	-1.1	0.925
Chronic bronchitis ± emphysema	817	0.486	847	0.659	0.738 (0.068)	-26.2	0.001
Chronic bronchitis ± emphysema with concomitant ICS	492	0.608	493	0.871	0.698 (0.077)	-30.2	0.001
Chronic bronchitis ± emphysema: no ICS	325	0.391	354	0.462	0.845 (0.140)	-15.5	0.310

Cough score at Week 0							
≥ 1 (average/day)	896	0.560	939	0.708	0.791 (0.067)	-20.9	0.006
< 1 (average/day)	395	0.523	385	0.508	1.030 (0.142)	3.0	0.830

Sputum score at Week 0							
≥ 1 (average/day)	829	0.576	862	0.700	0.822 (0.074)	-17.8	0.030
< 1 (average/day)	458	0.512	460	0.549	0.933 (0.113)	-6.7	0.565

Study completion status							
Completers	894	0.453	1011	0.573	0.790 (0.064)	-21	0.004
Non-completers	433	1.126	348	1.155	0.975 (0.113)	-2.5	0.826

Rates (per patient/year), Rate ratio and two-sided p-values (significance level 5%) are based on a Poisson regression model with the following factors and covariates: treatment, age, sex, smoking status, baseline post-bronchodilator FEV_1 _(% predicted), study, concomitant treatment with ICS and country pool (only for the overall population).

There were several subgroups in which the exacerbation rate appeared lower with roflumilast compared with placebo (Table 2), including patients with chronic bronchitis with or without emphysema (26.2% reduction in exacerbation rate vs placebo; p = 0.001). Other subgroups, such as current vs former smokers or those based on spirometrically defined COPD severity, showed no or little difference in the exacerbation rate with roflumilast. Patients receiving concomitant ICS experienced an 18.8% reduction in exacerbations compared with placebo (p = 0.014). Patients not receiving ICS exhibited no clinical benefit compared with placebo (Table 2). A significant reduction in exacerbation rate in favor of roflumilast was also seen in the subgroup of patients receiving concomitant short-acting anticholinergic treatment (18.3%, p = 0.012).

### Lung function

**Figure 3:**
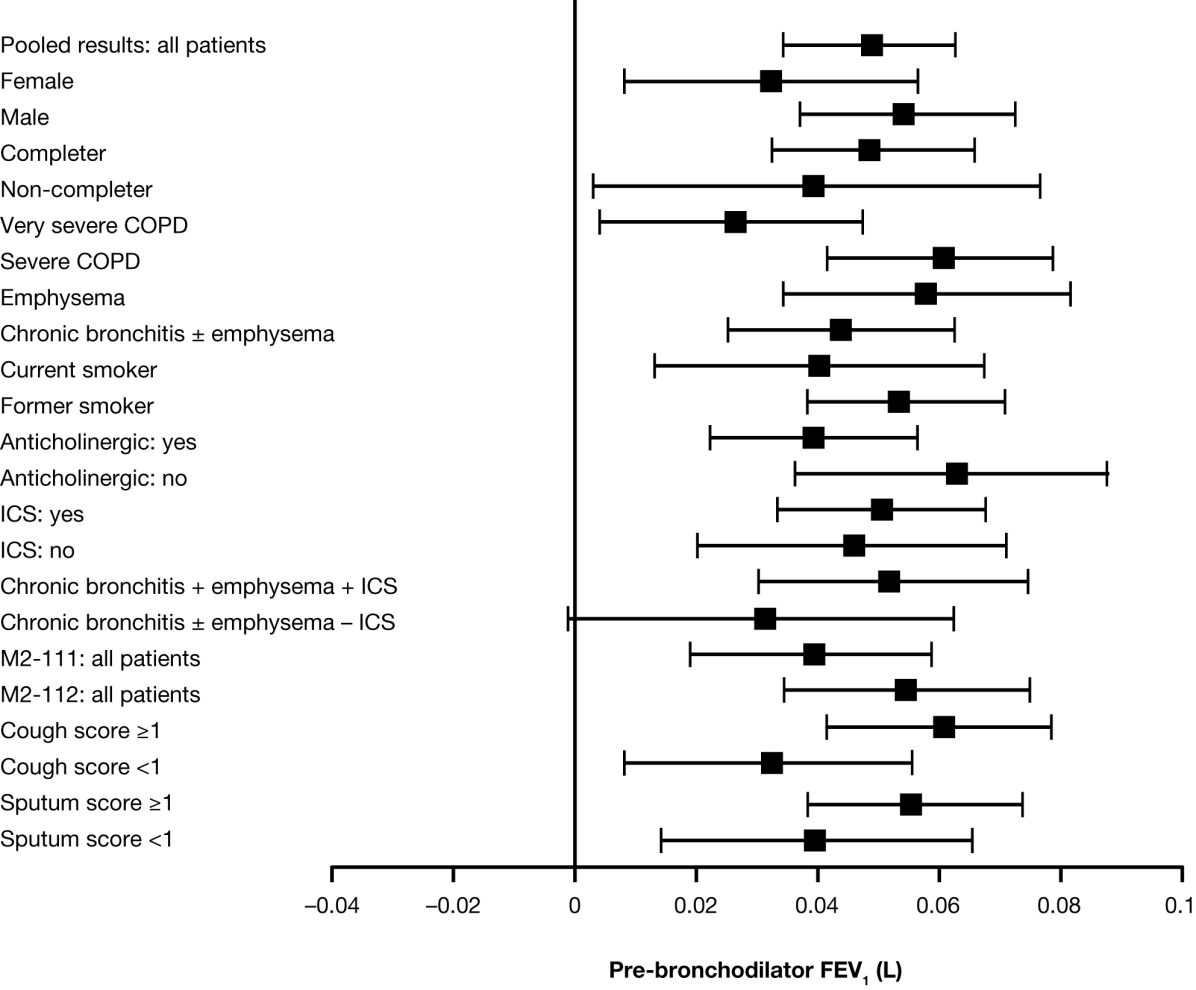
**Differences and 95% CIs between roflumilast and placebo for increase in pre-bronchodilator FEV**_**1 **_**(L) by patient subgroup**. Error bars represent 95% CIs.

Treatment with roflumilast resulted in significant improvement in pre-bronchodilator FEV_1 _compared with placebo. In the combined analysis, the improvement was evident at Week 4 (first measured time point) and maintained throughout the 52 weeks of the studies. After 52 weeks, the change in pre-bronchodilator FEV_1 _from baseline with roflumilast versus placebo was 51 mL (SE 7 mL, p < 0.0001), while the change in post-bronchodilator FEV_1 _with roflumilast vs placebo was 53 mL (SE 8 mL, p < 0.0001) (Figure 3; and see Additional file 1, Table S1). In contrast to the effect on exacerbations, roflumilast consistently showed a significant improvement compared with placebo in pre-bronchodilator FEV_1 _in all subgroups; the same was seen for post-bronchodilator FEV_1 _(see Additional file 1, Table S1). In the group of patients with COPD associated with chronic bronchitis or combined emphysema and chronic bronchitis, those patients receiving concomitant ICS showed a greater improvement from baseline with roflumilast vs placebo (see Additional file 1, Table S1).

### Health status

**Figure 4:**
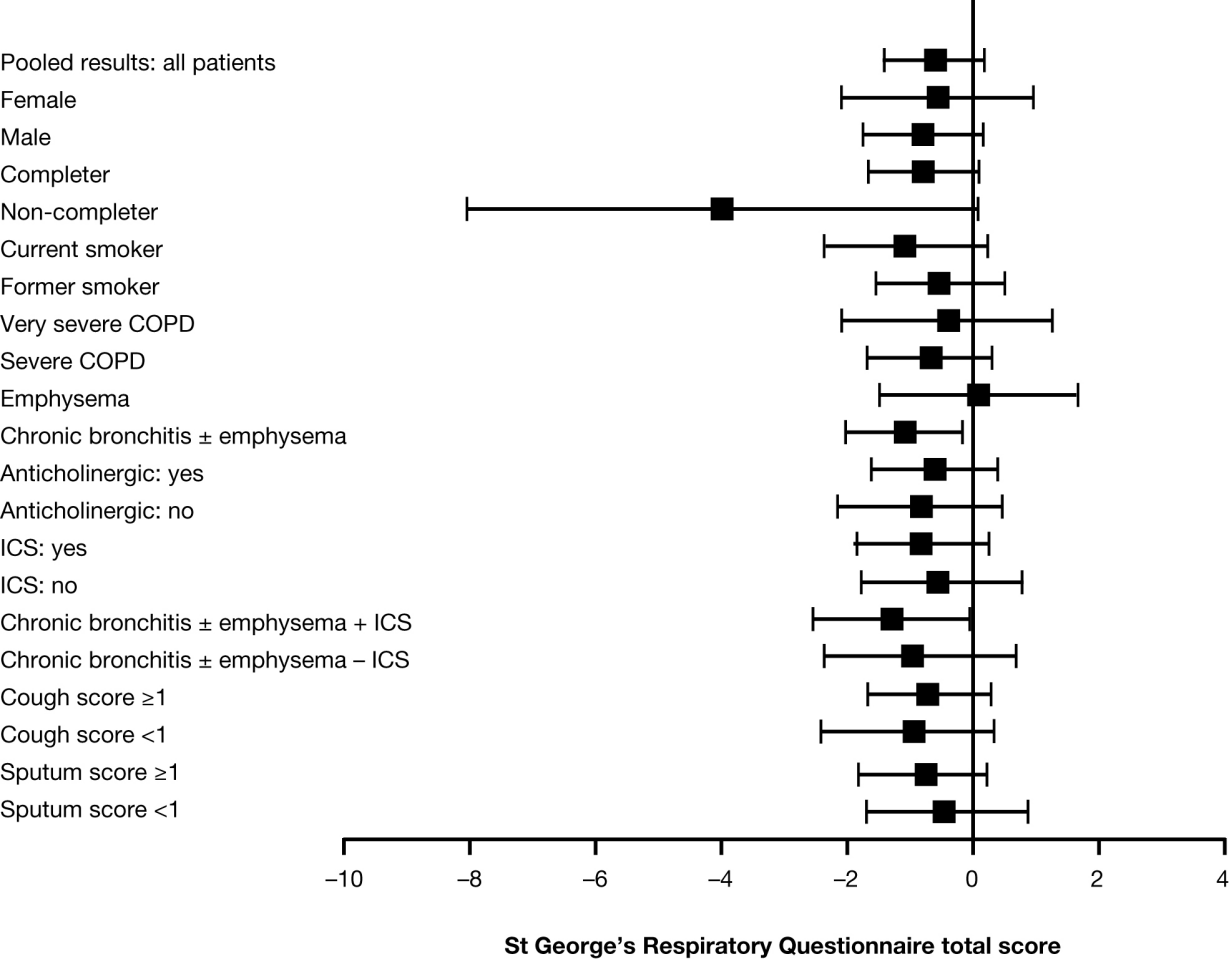
**Differences and 95% confidence intervals between roflumilast and placebo for changes in St George's Respiratory Questionnaire (SGRQ) total score by patient subgroup**. Error bars represent 95% CIs.

In the combined analysis, treatment with roflumilast resulted in no significant improvement in St George's Respiratory Questionnaire (SGRQ) total score compared with placebo. In contrast, in the subgroup analysis (Figure 4; and see Additional file 1, Table S2), a significant improvement in SGRQ total score was observed for individuals with chronic bronchitis (p = 0.0265). This difference was also evident in patients with chronic bronchitis who were concurrently treated with ICS (p = 0.0397).

### Safety

Adverse events were similar to those reported for roflumilast in previous studies (see Additional file 1, Appendix 3). Importantly, roflumilast (compared with placebo) was not associated with an increase in adverse events in the subgroups that experienced a greater reduction in exacerbations with roflumilast compared with placebo (Table 3; and see Additional file 1, Appendix 3). Concomitant ICS did not affect the adverse event profile of roflumilast.

**Table 3:** Adverse events

Subgroup	All patients		COPD history				CB ± emphysema and ICS treatment			
			Emphysema		CB ± emphysema		With ICS		Without ICS	
Treatment (n)	Rof (1327)	Pbo (1359)	Rof (352)	Pbo (413)	Rof (817)	Pbo (847)	Rof (492)	Pbo (493)	Rof (325)	Pbo (354)
*Adverse events, n (% of patients)*										
All adverse events	1081 (81.5)	1089 (80.1)	309 (87.8)	344 (83.3)	642 (78.6)	673 (79.5)	402 (81.7)	399 (80.9)	240 (73.8)	274 (77.4)
All serious adverse events	263 (19.8)	264 (19.4)	73 (20.7)	81 (19.6)	154 (18.8)	152 (17.9)	112 (22.8)	109 (22.1)	42 (12.9)	43 (12.1)
Adverse events related to study medication	285 (21.5)	113 (8.3)	91 (25.9)	39 (9.4)	134 (16.4)	67 (7.9)	77 (15.7)	35 (7.1)	57 (17.5)	32 (9.0)
Adverse events leading to study discontinuation	235 (17.7)	136 (10.0)	52 (14.8)	40 (9.7)	94 (11.5)	56 (6.6)	65 (13.2)	40 (8.1)	29 (8.9)	16 (4.5)

*Most common adverse events *(≥ *5% of patients in any treatment group), %*										
COPD exacerbation	42.9	48.0	45.5	47.7	43.0	48.5	49.8	54.4	32.6	40.4
Diarrhea	12.1	2.9	18.5	3.4	7.1	3.1	8.3	3.2	5.2	2.8
Nausea	6.0	1.5	8.0	1.9	4.4	1.3	4.7	1.0	4.0	1.7
Weight loss	7.5	2.8	11.9	4.1	6.1	2.5	5.3	1.6	7.4	3.7
Nasopharyngitis	6.8	7.4	7.7	8.2	7.5	7.7	6.5	7.3	8.9	8.2
Pneumonia	2.8	4.0	1.7	2.9	3.5	4.1	4.3	5.7	2.5	2.0
Upper respiratory tract infection	5.4	6.3	7.4	9.2	5.4	5.5	4.5	5.1	6.8	6.2
Headache	6.9	3.0	8.5	5.3	5.6	2.1	6.1	2.2	4.9	2.0
Influenza	4.4	4.0	5.4	5.3	4.4	3.7	4.5	2.2	4.3	5.6

Rof = roflumilast; pbo = placebo

## Discussion

PDE4 inhibitors have demonstrated an anti-inflammatory effect in animal models and patients with COPD [6,7]. In two previous 12-month studies, in patients with severe-to-very-severe COPD, roflumilast improved lung function, although neither study demonstrated a significant effect on exacerbations [10]. Given the pleiotropic effects of PDE4 inhibition [16], we hypothesized that a roflumilast effect could be present in specific subgroups of patients with COPD. In addition, exacerbation rates in the individual trials were lower than expected. Combining the datasets of the two studies improved statistical power and allowed definition of the patients more likely to respond to roflumilast. In the combined dataset, a significant effect of roflumilast was observed for the entire population but, importantly, the subgroup analysis showed a preferential effect in patients with chronic bronchitis or with high cough or sputum scores in the week prior to randomization, and in patients taking concomitant ICS or anticholinergics. These results suggested that it is possible to identify a subset of patients that is more likely to benefit from roflumilast with regard to reduced exacerbations.

In subjects with chronic bronchitis, this post-hoc, pooled analysis suggested a benefit of roflumilast on health status as measured by the SGRQ. The difference, compared with placebo, of -1.073 units did not achieve the conventional minimum important difference of 4 units, but was statistically significant and similar to differences seen between therapy in other 1-year trials [17]. This is consistent with the benefit in SGRQ resulting from the reduction in exacerbations.

Interestingly, roflumilast demonstrated a consistent effect on airflow, assessed as both pre- and post-bronchodilator FEV_1 _across all subgroups. There are several possibilities why the effect on exacerbations may be limited to a subset of patients. First, the subsets may identify those individuals at greater risk for exacerbations. A therapeutic benefit can be observed only if the individuals are at risk. Alternatively, as roflumilast can affect many aspects of the inflammatory response, it is possible that an anti-inflammatory effect, such as reduction in airway edema, may account for the improved airflow and a different mechanism accounts for the reduced exacerbations.

The effects seen with roflumilast in symptomatic patients and in patients with chronic bronchitis are comparable with those obtained by ICS/long-acting bronchodilator combination therapy [18-20]. The enhanced benefit of roflumilast in patients with chronic bronchitis is particularly interesting as this phenotype has been shown to be associated with serum markers indicative of increased systemic inflammation [21]. These patients are also at higher risk for mortality at a younger age [21]. The trend for a greater benefit in patients receiving concomitant ICS may be a marker of disease severity. This patient subgroup is at higher risk for exacerbations, indicated by the higher exacerbation rate in the placebo group in ICS-treated patients vs non ICS-treated patients (0.886 vs 0.460). That these individuals had been identified by their clinicians for treatment with ICS suggests that they were recognized as being at risk clinically and that further reductions in exacerbations and improved airflow were observed with roflumilast in this group suggests that a PDE4 inhibitor may add incremental value to ICS therapy.

Although the incidence of adverse events was comparable between treatment groups, there were more discontinuations due to adverse events with roflumilast compared with placebo. The majority of adverse events in both groups lasted less than 4 weeks and resolved with continued treatment. The incidence of treatment-related adverse events was low and similar to those reported previously [9,18]. These treatment-related events included diarrhea, nausea, and headache, which are all adverse events known to be associated with PDE4 inhibitors [22]. Weight loss was more frequent with roflumilast treatment. Several serious adverse events and deaths occurred, as would be expected in this patient population. The number of deaths was higher in the placebo group and most fatal events were related to COPD. A slightly higher incidence of adverse events and serious adverse events was seen in patients receiving ICS; this was seen in both the roflumilast and placebo groups. Oropharyngeal adverse events typically associated with ICS treatment, such as oral candidiasis, dysphonia, and pharyngitis, as well as pneumonia, were more frequently reported in patients treated with ICS, but there was no indication that roflumilast increased ICS-associated adverse events. Importantly, subjects with chronic bronchitis who were more likely to benefit from roflumilast did not experience an increased incidence of adverse events. On the contrary, there was a trend for these individuals to have fewer of the adverse events (nausea, diarrhea, and weight loss) that are associated with PDE4 inhibitors.

There are limitations to the pooled analysis presented in this manuscript, which includes both fully published and previously unpublished results. The post-hoc nature of the comparisons, particularly those in various subsets, must be interpreted with caution and serve principally as hypothesis generating. However, these results were used to design two additional randomized trials that specifically evaluated patients with severe COPD associated with chronic bronchitis, a group expected to be more likely to experience reductions in exacerbations with roflumilast. In this defined population, a significant beneficial effect of roflumilast compared with placebo in both lung function and exacerbation rate was observed in both studies [15]. In this context, the sequence of studies is crucial. Following a phase 2 trial that showed promising results [9], two 'conventional' 12-month phase 2 trials (Study M2-111, reported here for the first time, and M2-112 [10]) were conducted, both of which showed improvements in FEV_1 _but demonstrated only a trend toward exacerbation reduction. The pooled analysis presented here demonstrated that a subset of the COPD population appeared to account for all the benefit with regard to exacerbations. This 'hypothesis' formed the basis of two subsequent trials [15] which demonstrated the efficacy of roflumilast for exacerbation reduction in this subset.

Novel therapies for COPD are urgently needed [11]. The current manuscript describes the successful use of a strategy for identification of a responding subset from clinical trial data that was then confirmed in two prospective, randomized, placebo-controlled clinical trials. At present, segmentation of meaningful sub-populations of COPD patients is difficult, although several large observational studies are addressing this question. The current study demonstrates that this goal can also be achieved by post-hoc analysis of responses to a clinical intervention.

## Conclusions

This post-hoc, pooled analysis of two large-scale trials in patients with severe and very severe COPD showed a significant reduction in exacerbations with roflumilast treatment and identified a subgroup of patients who are most likely to benefit from treatment with roflumilast, namely those patients with chronic bronchitis. In addition there was a greater effect in those patients taking concomitant ICS. Identification of a subgroup of patients more likely to respond to therapy is consistent with the concept that the COPD population includes multiple phenotypes and is a step towards personalized medicine, matching therapy to phenotype [11,23,24]. Importantly, identification of a responding subset can facilitate drug development by increasing the ability of clinical trials to show a benefit. In this regard, the analysis presented in the current report was used to design subsequent clinical trials that have demonstrated the clinical efficacy of roflumilast in reducing COPD exacerbations. This is the first time such an approach has been used successfully to aid a drug development program in COPD.

## Abbreviations

ANCOVA: Analysis of covariance; COPD: chronic obstructive pulmonary disease; FEV_1_: forced expiratory volume in 1 second; ICS: inhaled corticosteroids; PDE4: phosphodiesterase 4; SD: standard deviation; SE: standard error; SGRQ: St George's Respiratory Questionnaire.

## Competing interests

SIR has served on advisory boards and as a consultant for Almirall Prodesfarma, Aradigm Corporation; AstraZeneca, Boehringer Ingelheim, Defined Health, Eaton Associates, GlaxoSmithKline, MEDACorp, Mpex Pharmaceuticals, Novartis, Nycomed, Otsuka Pharmaceutical, Pfizer, Pulmatrix, Theravance, United BioSource Corporation, Uptake Medical, and VantagePoint. He has served as a speaker or a member of a speaker's bureau for: AstraZeneca, Novartis, Network for Continuing Education, Pfizer, and SOMA. He has also received research funding from AstraZeneca, BioMarck, Centocor, Novartis, and Nycomed.

PMAC has served on advisory boards for AstraZeneca, GlaxoSmithKline, Nycomed, and Novartis. He has received research funding from GlaxoSmithKline, Nycomed, and Boehringer Ingelheim, and has spoken at meetings supported by AstraZeneca, GlaxoSmithKline, and Nycomed.

FJM has been a member of advisory boards for GlaxoSmithKline, Schering Plough, Novartis, Nycomed, Genzyme, Forest/Almirall, MedImmune, AstraZeneca, Potomac, Bayer, Elan, Talecris, and Roche. He has been on the speaker's bureau for Boehringer Ingelheim, GlaxoSmithKline, France Foundation, MedEd, NACE, and AstraZeneca. He has also been a member of steering committees for studies supported by Altana/Nycomed, GlaxoSmithKline, Gilead, Actelion, Johnson/Johnson, Mpex, UCB, and the National Institutes of Health. He has been an investigator in trials supported by Boehringer Ingelheim and Actelion.

UMG and DB are employees of Nycomed GmbH, Konstanz, Germany.

## Authors' contributions

SIR contributed to the conception and design of these studies, the acquisition of study data, and the analysis and interpretation of these data. He was fully involved in the drafting and revision of this manuscript, and provided final approval of its content ahead of submission. PMAC contributed to the conception and design of these studies, the acquisition of study data, and the analysis and interpretation of these data. He was fully involved in the drafting and revision of this manuscript, and provided final approval of its content ahead of submission. U-MG contributed to the conception and design of these studies, the acquisition of study data, and the analysis and interpretation of these data. He was fully involved in the drafting and revision of this manuscript, and provided final approval of its content ahead of submission. He had full access to all of the data in the study and he takes full responsibility for the integrity of all of the data and the accuracy of the data analysis, including and especially any adverse effects. DB contributed to the conception and design of these studies, the acquisition of study data, and the analysis and interpretation of these data. He was fully involved in the drafting and revision of this manuscript, and provided final approval of its content ahead of submission. FJM contributed to the conception and design of these studies, as well as the analysis and interpretation of these data. He was fully involved in the drafting and revision of this manuscript, and provided final approval of its content ahead of submission.

## Supplementary Material

Additional file 1 **Appendices 1-3, Table S1, Table S2, and Figure S1**. Appendix 1: Trial design; Appendix 2: IRB approval; Appendix 3: Adverse events; Table S1: Lung function results summary table (change in lung function variable after 52 Weeks compared with baseline); Table S2: St George's Respiratory Questionnaire (SGRQ) total score: change after 52 Weeks compared with baseline; Figure S1: Trial profile of M2-111. Click here for file

Additional file 2 **List of investigators for Studies M2-111 and M2-112**. M2-111 investigators; M2-112 investigators. Click here for file
