# Supplementary material for: Reduction of exacerbations by the PDE4 inhibitor roflumilast - the importance of defining different subsets of patients with COPD
Source: Respir Res. 2011 Jan 27;12(1):18. doi: 10.1186/1465-9921-12-18 (PMC3040135; doi:10.1186/1465-9921-12-18)
Supplement: Additional file 1 — Appendices 1-3, Table S1, Table S2, and Figure S1. Appendix 1: Trial design; Appendix 2: IRB approval; Appendix 3: Adverse events; Table S1: Lung function results summary table (change in lung function variable after 52 Weeks compared with baseline); Table S2: St George's Respiratory Questionnaire (SGRQ) total score: change after 52 Weeks compared with baseline; Figure S1: Trial profile of M2-111. [file 1465-9921-12-18-S1.DOC]

# Additional File 1

# Exacerbation reduction by roflumilast – importance of defining different subsets of patients with COPD

Stephen I Rennard^1^, Peter MA Calverley^2^, Udo M Goehring^3^, Dirk Bredenbröker^3^, Fernando J Martinez^4^

^1^Nebraska Medical Center, Omaha, USA, ^2^University Hospital Aintree, Liverpool, UK, ^3^Nycomed GmbH, Konstanz, Germany, ^4^University of Michigan Medical Center, Ann Arbor, USA

**Contents**

**Appendix 1: Trial design**

**Appendix 2: IRB approval**

**Appendix 3: Adverse events**

**Table S1: Lung function results summary table (change in lung function variable after 52 Weeks compared with baseline)**

**Table S2: St George’s Respiratory Questionnaire (SGRQ) total score: change after 52 Weeks compared with baseline**

**Figure S1: Trial profile of M2-111**

**Appendix 1**

**Trial designs**

M2-111 and M2-112 were randomized, double-blind, placebo-controlled, parallel-group studies recruiting outpatients ≥ 40 years of age meeting established diagnostic criteria for chronic obstructive pulmonary disease (COPD) who were clinically stable, with no moderate or severe exacerbations and no change in COPD treatment within 4 weeks of baseline. After a 4-week, single-blind run-in period during which patients received placebo, patients were randomly assigned (1:1 ratio) to receive either roflumilast 500 µg or placebo administered once daily in the morning for 52 weeks. Treatment with inhaled corticosteroids (≤ 2000 μg beclomethasone dipropionate or equivalent) and short-acting anticholinergics was allowed if used before study entry. All patients were allowed salbutamol as rescue medication, but long-acting muscarinic antagonists and long-acting β_2_-agonists were not allowed. The study protocols are registered on www.ClinicalTrials.gov (NCT00076089 [M2-111] and NCT00430729 [M2-112]). In both studies, moderate exacerbations were defined as symptomatic deteriorations treated with systemic corticosteroids and/or antibiotics while severe events were those requiring hospitalization. Health status was assessed using the St George’s Respiratory Questionnaire (SGRQ) at entry, at 2 and 4 weeks after the start of the run-in period, and at 12, 28 and 52 weeks during the treatment period. In addition, the SGRQ was also completed at weeks 36 and 44 during the treatment period for study M2-112.

Although the pooled analysis was pre-specified in the statistical analysis plans of both studies prior to unblinding of either study, the statistical analysis plan of the pooled analysis was finalized only after the first study had been completed and unblinded. Consequently, results from the pooled analysis are not designed to provide definitive conclusions, but rather as exploratory studies designed to identify subpopulations that may be appropriate to test a more refined hypothesis.

**Appendix 2**

**IRB approvals**

**List of Independent Ethics Committees (along with the investigators involved)**

**BY217/M2-111 OPUS Study**

| **Ethics committee** | **Investigator(s)** | **Center No.** | **IRB Approval No.** |
| --- | --- | --- | --- |
| **Canada** |  |  |  |
| Research Ethics Board of the MUHC Montreal Chest Institute of the Royal Victoria Hospital 3650 St. Urbain CA-Montreal, Quebec H2X 2P4 | Dr J. Bourbeau | 2077 | # 03-38 |
| Institutional Review Board Services 14845-6 Yonge Street, Suite 328 CA-Aurora, Ontario, L4G 6H8 | Dr B. Carlson   Dr V. Chan   Dr A. Dowell   Dr S. Henein   Dr J. Lenis   Dr A Nayar | 5040   4850   4197   4834   3989   4839 | No IRB Project # provided Date of vote: 27.08.2003 No IRB Project # provided Date of vote: 27.08.2003 No IRB Project # provided Date of vote: 27.08.2003 No IRB Project # provided Date of vote: 27.08.2003 No IRB Project # provided Date of vote: 27.08.2003 No IRB Project # provided Date of vote: 27.08.2003 |
| Research Ethics Board, Office of Research Administration, St. Michael's Hospital 30 Bond Streeet CA-Toronto, Ontario M5B 1W8 | Prof. Dr V. Hoffstein | 4836 | REB # 03-293 |
| Concordia Hospital Concordia Ethics Committee 1095 Concordia Avenue CA-Winnipeg, Manitoba R2K 3S8 | Dr L. Homik | 4313 | No IRB Project # provided Date of vote: 05.09.2003 |
| Sudbury Regional Hospital Research Ethics Committee Laurentian Site 41, chemin du lac CA-Sudbury, Ontario P3E 5J1 | Dr R. Dhatt | 5199 | No IRB Project # provided Date of vote: 27.08.2003 |
| Human Research Ethics Committee Fleurimont Hospital 2001, 12^th^ Avenue North CA-Fleurimont, Quebec J1M 5N4 | Prof. Dr P. Larivée | 2712 | #03-67 (OPUS) |
| Capital Health Research Ethics Board Centre for Clinical Research 5790 University Avenue CA-Halifax, Nova Scotia  B3H 1V7 | Dr A. McIvor | 3136 | # CDHA-RS  2003-218 |
| Hopital du Saint-Sacrement Comite d’ethique  1050 chemin Sainte-Foy CA-Sainte-Foy Quebec G1S 4L8 | Dr M. Rouleau | 3994 | # DR-002-1059 |
| **France** |  |  |  |
| CCPPRB de Lyon Hopital Hötel Dieu-Porte 16 Place de l'Hopital F-69288 Lyon Cedex 02 | Responsible for all French investigators | 4883 4264 2311 2300 2190 3998 3550 2099 4882 2310 1647 1881 | 2003-064/217A |
| **Germany** |  |  |  |
| Ethikkommission der Landesärztekammer Baden-Württemberg Jahnstr. 40 D-70597 Stuttgart | responsible for all German investigators | 3980  3318  3980 | Ref. No. 111-03 |
| Ärztekammer des Saarlandes Ethikkommission Faktoreistr. 4 D-66111 Saarbrücken | Dr Th. Dapper | 5062 | Ref. no. 211/03 |
| Ärztekammer Berlin Ethikkommission Friedrichstr. 16 D-10969 Berlin | Dr J. Eller Prof. Dr G. Kunkel Dr J. Pettenkofer | 3075 4331 2977 | Eth-837-305/03 |
| Bayerische Landesärztekammer Ethikkommission  Mühlbauerstr. 16 D-81677 München | Dr W. Hübner Dr N. Reinholz Dr K.-D. Rost Dr H. Steffen Dr J. Weöres | 5061 5059 1456 1436 5060 | Reference 7/03214 |
| Ärztekammer Nordrhein Ethikkommission Tersteegenstr. 9 D-40474 Düsseldorf | Dr Th. Ginko | 3365 | Ctd. No. 2003332 |
| Ärztekammer Sachsen-Anhalt  Ethikkommission Bez.-Geschäftsstelle Halle Am Kirchtor 9 D-06108 Halle  Ethikkommission der Otto-von-Guericke Universität Magdeburg Leipziger Straße 44 39120 Magdeburg | Dr A. Piecyk | 4795 | Sign 136/03  Sign 184/03 |
| Landesärztekammer Hessen Ethikkommission Im Vogelgesang 3 D-60488 Frankfurt | Dr H. Tauth | 1526 | E 282/2003MC |
| **Poland** |  |  |  |
| Bioethics Committee at Military Medical Chamber Ul Szaserow 128 PL-00-909 Warszawa | Dr M. Bizunowicz-Artecka  Dr K. Folcik  Dr B. Klimkiewicz | 5312    5311  4379 | Resolution No: 26/2004    Resolution No: 22/2004  Resolution No: 20/2004 |
| Bioethics Committee of the Silesian Doctors Chamber in Katowice Ul. Grazynskiego 49A PL-40-126 Katowice | Dr J. Cieslicki | 5310 | Resolution No: 20/2004 |
| Bioethics Committee at the Regional Medical Chamber Ul Norwida 4 PL-81-434 Gdynia | Dr E. Gross-Tyrkin  Dr P. Miekus   Dr M. Piepiorka | 4557   4559   4556 | Resolution No: 288/2004  Resolution No: 287/2004  Resolution No: 286/2004 |
| Bioethics Committee Medical University Al. Kosciuszki 4 PL-90-419 Lodz | Prof. Dr D. Nowak   Prof. Dr I. Grzelewska-Rzymowska | 4561   4560 | Resolution No: RNN/76/04/KE  Decision No: RNN/110/04/KE |
| Bioethics Committee of District Council of Doctors  Ul. Chmielna 4 PL-20-079 Lublin | Dr E. Trebas-Pietras | 4564 | Resolution No. 258/2004/KB |

| **South Africa** |  |  |  |
| --- | --- | --- | --- |
| Pharma Ethics (PTY.) Ltd. 123 Amcor Road RSA- Lyttleton Manor 0157 | Responsible for all South African investigators | 1969 3116 4021 4283 4284 4804 4805 4808 4810 4858 4859 5087 5573 | Ref. no. 03090844 |
| University of Cape Town Research Ethics Committee Faculty of Health Sciences Old Main Building  Groote Schuur Hospital RSA-Observatory, 7925 | Prof. Dr E. Bateman | 3397 | Date of vote: 01.08.2003 |
| Committee of Pharmaceutical Trials of the University of Stellenbosch PO Box 19063 RSA-Tygerberg 7505 | Prof. Dr E. Irusen | 4806 | Date of vote: 27.01.2004 |
| **United States of America** |  |  |  |
| McGuire Institutional Review Board 1201 Broad Rock Boulevard USA-Richmond, VA 23249 | Dr O. Abdullah | 5257 | ID: 00954 Prom#0010 |
| Schulman Associates IRB (SAIRB) 4290 Glendale Milford Road USA-Cincinnati, OH 45242 | Dr R. Abrahams Dr M. Alter Dr S. Babazedah Dr A. Baker Dr St. Basheda Dr R. Bell Dr R. Benkert Dr D. Bernstein Dr G. Blyden Dr D. Cardona Dr B. Charous Dr P. Chervinsky Dr J. Cohn Dr A. Dahdul Dr A. DeGraff Dr E. Diamond Dr L. Dunn Dr St. Eaton Dr W. Ellison Dr J. Enden Dr J. Epstein Dr D. Fried Dr R. Gilman Dr M. Gotfried Dr V. Greiff Dr D. Hill Dr T. Horiuchi Dr St. Horner Dr N. Horning Dr J. Hoyt Dr M. Jacobs Dr J. Jayne Dr Th. Kaelin Dr F. Kahn Dr R. Kahn Dr H. Kaiser Dr M. Kaye Dr E. Kerwin Dr Ph. Korenblat Dr St. Kreitzer Dr J. Lampasso Dr R. Larimer Dr E. Lisberg Dr D. Lorch Dr Ph. Marcus Dr C. Mello Dr D. Meyer Dr Morganroth Dr A. Nayak Dr Th. O'Barr Dr P. Parikh Dr V. Patel Dr J. Pearle Dr D. Puryear Dr J. Quesada Dr R. Rab-Hasan Dr B. Rankin Dr J. Rehm Dr L. Repsher Dr J. Rhudy Dr A. Rooklin Dr P. Sachs Dr J. Schelbar Dr E. Schenkel Dr J. Sibille Dr Th. Siler Dr A. Silverthorn Dr W. Sinclair Dr J. Singh Dr B. Snyder Dr Spangenthal Dr I. Spirn Dr B. Streit Dr R. Sussman Dr J. Thiele Dr R. Tidman Dr T. Warren Dr S. Weakley Dr P. Weinberg Dr J. Winder Dr J. Wolfe Dr R. Wolfe | 5214 5626 4142 5245 5188 3565 3451 2917 5843 5265 5189 4821 5595 4151 3472 3500 4171 5594 5207 4077 5269 5270 5272 5273 4081 4771 5317 5278 2846 5254 5190 5611 5191 5279 5208 3412 5602 3854 3433 5255 5233 5286 4166 2831 5248 5447 5192 2789 4789 5290 5209 5296 5299 5603 5212 5256 3446 5301 5250 5194 5251 5252 4779 2915 5195 3479 4087 3480 5306 5841 5307 4169 4510 3859 5442 5253 5313 3464 5314 3468 3426 4185 | 2003-4527-0 |
| Mt. Sinai Medical Center Institutional Review Board 4300 Alton Road USA-Miami Beach, FL 33140 | Prof. Dr T. Ahmed | 5258 | Prot # 03-91-H-12 |
| Western Institutional Review Board 3537 7^th^ Avenue SW USA-Olympia, WA 98502 | Dr W. Bailey | 5261 | Study # 1056398;  WIRB Pro # 20040126;  Invest # 105304;  Wo # 1-259141-1 |
| Newark Campus - UMDNJ New Jersey Medical School 150 Bergen Street USA-Newark, NJ 07103 | Dr L. Bielory | 5262 | IRB Protocol # M-071-2004 |
| Wake Forest University Health Sciences Institutional Review Board Medical Center Boulevard USA-Winston-Salem, NC 27157 | Dr E. Bleecker | 5263 | Human Protocol # BG04-019 |
| Birmingham VA Institutional Review Board Department of Veterans Affairs Medical Center 700 South 19^th^ Street VA Research Service (151) USA-Birmingham, AL 35233 | Prof. Dr J. Cooper | 5266 | ID: 01124 |
| Human Subjects Protection Office The Penn State University The Milton S. Hershey Medical Center 500 University Drive USA-Hershey, PA 17033 | Prof. Dr T. Craig | 3566 | IRB # 2003-381 |
| Committee on Human Research  University of Vermont 231 Rowell Building USA-Burlington, VT 05405 | Prof. Dr G. Davis | 5267 | IRB # 00000485 |
| University of Kentucky Institutional Review Board 315 Kinkead Hall USA-Lexington, KY 40506-0057 | Dr D. Doherty | 2817 | IRB # 04-0204-F3R |
| The Committee of the Protection of Rights of Human Subjects University of North Carolina at Chapel Hill CB # 7097, Medical School Building 52 USA-Chapel Hill, NC 27599-7097 | Dr J. Donohue | 5268 | IRB Study # 04-PUL-125 |
| McGuire Institutional Review Board 1201 Broad Rock Boulevard USA-Richmond, VA 23249 | Dr Th. Ferro | 5298 | ID 00949 |
| Norwalk Hospital Institutional Review Board Maple Street USA-Norwalk, CT 06850 | Dr J. Fine | 5521 | IRB # 04-20 |
| Human Studies Subcommittee 5^th^ Avenue and Roosevelt Road USA-Hines, FL 60141 | Dr A. Fulambarker  Dr N. Gross | 5271  5274 | IRB # 03-125  IRB # 04-009 |
| Institutional Review Board for Human Subject Research for Baylor College of Medicine and Affiliated Hospitals One Baylor Plaza, Room 600D USA-Houston, TX 77030 | Prof. Dr K. Guntupalli | 3043 | H15136 |
| University of Arizona Human Subjects Protection Committee 1350 N. Vine Avenue USA-Tucson, AZ 85724-5137 | Prof. Dr M. Habib | 5275 | Study # 1056218,  WIRB Pro # 20040126,  Invest # 69465,  Wo # 1-249658-1 |
| Medical College of Ohio Institutional Review Board 3000 Arlington Ave. USA-Toledo, OH 43614 | Dr J. Hammersley | 5276 | IRB # 104580 |
| Human Research Advisory Committee University of Arkansas for Medical Sciences 4301 West Markham Street # 636 USA-Little Rock, AK 72205 | Prof. Dr Ch. Hiller | 5277 | FWA 00001119,  Record: 28831 |
| Western Institutional Review Board 3535 Seventh Avenue USA-Olympia, WA 98508-2029 | Dr J. Ilowite | 3849 | Study # 1056218,  WIRB Pro # 20040126,  Invest # 69465,  Wo # 1-249658-1 |
| Biomedical Research Alliance of New York, LLC Institutional Review Board 225 Community Drive USA-Great Neck, NY 11021 | Dr J. Karpel | 5280 | IRB # 03A-02-208-03 |
| Institutional Review Board Temple University 3425 Carlisle Street USA-Philadelphia | Prof. Dr St. Kelsen | 5281 | IRB Prot # 4347 |
| Committee on Use of Human Subjects 1430 Tulane Avenue, TW 36 USA-New Orleans, LA 70112 | Prof. Dr K. Kovitz | 5441 | KO0222 |
| University of Nevada-Reno Office of Human Research Protection UNR Institutional Review Board 205 Ross Hall, 331 USA-Reno, NV 89557-0246 | Dr P. Krumpe | 5285 | Protocol B03/04-15 |
| Administrative Panel of Human Subjects in Medical Research 1215 Welch Road USA-Stanford, CA 94305-5401 | Dr W. Kuschner | 2822 | Prot ID: 79911,  IRB # 350 (panel 3) |
| University of Oklahoma Health Sciences Center Institutional Review Board 1000 S.L. Young Boulevard, Library 176 USA-Oklahoma, OK 73117 | Prof. Dr D. Levin | 5287 | IRB # 11419 |
| Research and Development Human Studies Subcommittee VA Greater Los Angeles Healthcare System 16111 Plummer Street USA-Sepulveda, CA 91343 | Dr M. Littner | 5291 | PCC 2004-010126 |
| Committee for the Protection of Human Subjects Office of Grants and Contracts 11 Rope Ferry Road USA-Hanover, NH 03755-1404 | Prof. Dr D. Mahler | 5292 | CPHS # 16710 |
| Human Studies Subcommittee VA Long Beach Health Care System 5901 E. 7^th^ Street USA-Long Beach, CA 90822 | Prof. Dr C. Mahutte | 5293 | MIRB: 00511 |
| Salem V.A.M.C. Institutional Review Board 1970 Roanoke Blvd. (151) USA-Salem, VA 24153 | Dr Th. Martin | 5294 | TJM 0012 |
| Vanderbilt University Institutional Review Board D-3232 Medical Center North USA-Nashville, TN 37232-2598 | Prof. Dr J. Murray | 5295 | IRB # 040163 |
| National Jewish Medical and Research Center Institutional Review Board 1400 Jackson Street USA-Denver, CO 80206 | Prof. Dr H. Nelson | 3045 | HS-1805 |
| Institutional Review Board University of Nebraska Medical Center 987830 Nebraska Medical Center USA-Omaha, Nebraska 68198-7830 | Dr St. Rennard | 5302 | IRB # 445-03 |
| Minneapolis VA Medical Center IRB Human Stides Subcommittee Research 151 One Vetrans Drive USA-Minneapolis, MN 55417 | Dr K. Rice | 5303 | Protocol # 3377A |
| UCSD Human Subjects Committee 9500 Gilman Drive USA-LaJolla, CA 92093-0052 | Prof. Dr A. Ries | 5304 | Project # 040065 |
| Medical University of South Carolina Office of Research Integrity and Risk Protection 165 Cannon Street USA-Charleston, SC 29425 | Prof. Dr St. Sahn | 3865 | HR # 11299 |
| Mount Sinai School of Medicine Institutional Review Board One Gustave L. levy Place, Box 1075 East Building, 4-78 USA-New York, NY 10029-6574 | Prof. Dr N. Schachter | 2739 | 04-034 (0001) 01 ME |
| Institutional Review Board for Human Subject Research at Baylor College of Medicine and Affiliated Hospitals Baylor College of Medicine One Baylor Plaza USA-Houston, TX 77030 | Dr A. Sharafkhaneh | 5305 | H-14919 |
| UCLA Office for the Protection of Research Subjects Medical Institutional Review Board 10945 le Conte Avenue Ubberroth Bldg, Suite 2107 USA-Los Angeles, CA 90095 | Prof. Dr D. Tashkin | 5308 | IRB # 03-12-019-03 |
| Harbor-UCLA Research and Education Institute Institutional Review Board 1124 W. Carson Street USA-Torrance, CA 90502 | Dr J. Vintch | 5309 | Research Project # 11721-01 |
| Human Subject Research Office Jackson Medical Towers East 1500 NW 12 Avenue USA-Miami, FL 33136 | Dr A. Wanner | 3484 | Protocol # 2003-0914A |
| Sharp Healthcare Institutional Review Board 8695 Spectrum Center Blvd. USA-San Diego, CA 92123 | Dr D. Wilms | 5315 | IRB # 040492 |
| University of Health Sciences Institutional Review Board 1750 Independence Avenue USA-Kansas City, MO 64106 | Prof. Dr S. Willsie | 4754 | Study # 03-18 |

**List of Independent Ethics Committees (along with the investigators involved)**

**BY217/M2-112 RATIO Study**

| **Ethics committee** | **Investigator(s)** | **Center No.** | **IRB Approval No.** |
| --- | --- | --- | --- |
| **Austria** |  |  |  |
| Ethikkommission Oberösterreich Wagner-Jauregg-Weg 15 A-4020 Linz | Dr J. Grillenberger Dr G. Holub Dr J. Würtz Dr W. Höller | 2899 2901 4279 4278 | EK Nr.145 EK Nr.145 EK Nr.145 EK Nr.145 |
| Ethikkommission für das Land Niederösterreich am Sitz des Amtes der NÖ Landesregierung Landhausplatz 1, Haus 15 B A-3109 St. Pölten | Dr W. Pohl | 2904 | GS 4-EK-AMG/105 |
| Ethikkommission Salzburg Sebastian-Stief-Gasse 2 A-5010 Salzburg | Dr. M. Sweilem | 2906 | E-Nr.: 439 |
| Ethikkommission der Stadt Wien  Magistratsabteilung 15 - Gesundheitswesen - Schottenring 24 A-1010 Wien | Dr H. Zwick | 2377 | GZ: EK 02-113-1202 |
| **Australia** |  |  |  |
| South Western Sydney Area Health Service Human Research Ethics Committee Commercial Services Building Eastern Campus, Liverpool Elizabeth Street AU-Liverpool 2170, NSW | Dr H. Crawford | 4320 | Ref. no. 03/029 |
| Repatriation General Hospital Research & Ethics Committee Repatriation General Hospital Daws Road AU-Daw Park 5041, SA | Dr P. Frith | 3913 | Ref. no. 68/02 |
| Royal Adelaide Hospital Human Research Ethics Committee Office of the Deputy Vice-Chancellor The University of Adelaide AU-Adelaide 5005, SA | Dr M. Holmes | 4015 | Ref. no. 021112 |
| Central Sydney Area Health Service Research Ethics Committee (RPAH Zone) Level 8, Building 14 Royal Prince Alfred Hospital Missenden Road AU-Camperdown 2050, NSW | Dr Ch. Jenkins | 3910 | Ref. no. X02-0281/X02-0015 |
| Western Sydney Area Health Service Human Research Ethics Committee Research Office Westmead Hospital AU-Westmead 2145, NSW | Dr P. Middleton | 4321 | Ref. no. 2003/2/4.2 (1581) |
| Princess Alexandra Hospital  Human Research Ethics Committee Ipswich Road AU-Woolloongabba 4102, QLD | Dr Ch. Mitchell | 3918 | Ref. no. 164/02 |
| The Central Sydney Area Health Service Human Research Ethics Committee Concord Repatriation General Hospital Hospital Road AU-Concord 2139, NSW | Dr M. Peters | 4019 | Ref. no. CH 62/6/2002-100-Peters |
| Royal Melbourne Hospital  Research Foundation & Ethics Committee Charles Connibere Building Grattan Street AU-Parkville 3050, Vic | Dr A. Rubinfeld | 3897 | Ref. no. 2002.203 |
| North Western Adelaide Health Service Ethics of Human Research Committee 28 Woodville Road AU-Woodville 5011, South SA | Dr A.M. Southcott | 3915 | Ref. no. 122/02 |
| Sir Charles Gairdner Hospital Human Research Ethics Committee Hospital Avenue AU-Nedlands 6009, WA | Prof. Ph. Thompson | 3916 | Ref. no. 2002-135 |
| **Canada** |  |  |  |
| Institutional Review Board Services 14845-6 Yonge Street, Suite 328 CA-Aurora, ON L4 6H8 | Dr M. Alexander   Dr E. Amer   Dr D. Dattani   Dr Th. Fera   Dr J. Hebert   Dr F. Jardine   Dr R. Luton   Dr R. Maleki-Yazdi   Dr J. Muscedere   Dr B. Ramjattan   Dr R. Somani | 4308   3982   4310   2085   4312   4314   3990   3273   2754   4318   4319 | No IRB Project # provided Date of vote: 18.10.2002 No IRB Project # provided Date of vote: 18.10.2002 No IRB Project # provided Date of vote: 18.10.2002 No IRB Project # provided Date of vote: 18.10.2002 No IRB Project # provided Date of vote: 06.11.2002 No IRB Project # provided Date of vote: 05.11.2002 No IRB Project # provided Date of vote: 18.10.2002 No IRB Project # provided Date of vote: 27.11.2002 No IRB Project # provided Date of vote: 18.10.2002 No IRB Project # provided Date of vote: 18.10.2002 No IRB Project # provided Date of vote: 18.10.2002 |
| University of Calgary Office of Medical Bioethics Room 93, Heritage Medical Research Building 3330 Hospital Drive NW CA-Calgary, T2N 4N1 | Dr G. Ford | 4311 | # 16788 |
| The Ottawa Hospital Research Ethics Board 737 Parkdale Avenue, Room 615 CA-Ottawa, K1Y 4E9 Ontario | Dr R. Hodder | 417 | 2002518-01H |
| Research Ethics Committee of Laval Hospital 2725 Chemin Sainte-Foy CA-Sainte Foy, QC G1V 4G5 | Dr F. Maltais | 2071 | # 908 |
| Concordia Hospital Ethics Committee Concordia Hospital 1095 Concordia Avenue CA-Winnipeg, MB R2K 3S8 | Dr L. Homik | 4313 | No IRB Project # provided Date of vote: 08.10.2002 |
| University of Saskatchewan Biomedical Research Ethics Board 117 Science Place CA-Saskatoon, SK S7N 5C8 | Dr D. Marciniuk | 4315 | # BMC02-685 |
| Capital Health Research Ethics Board Center for Clinical Research 5790 University Avenue CA-Halifax, B3H 1V7 Nova Scotia | Dr A. McIvor | 2751 | # CDHA-RS-2002-286 |
| Research Ethics Board Sunnybrook & Women’s College Health Sciences Centre The Research Building  2075 Bayview Avenue, Room S1 33 CA-Toronto, ON M4N 3M5 | Dr Sh. Mintz | 2535 | # 385-2002 |
| College of Physicians & Surgeons of Alberta Research Ethics Board 900 Manulife Place 10180-101 Street CA-Edmonton, T5J 4P8 Alberta | Dr W. Ramesh | 4316 | # REC-870 |
| CHUM Centre de Recherche Comité d’éthique de la recherche, Hotel-Dieu 3840, rue Saint-Urbain Pavillon Masson, Porte 8107B CA-Montreal, QC H2W 1T7 | Dr P. Renzi | 4317 | # HD 02.065 |
| Research Ethics Office Jewish General Hospital CA-Montreal, Quebec | Dr D. Small | 3124 | # 02-112 |
| **France** |  |  |  |
| Comité Consultatif de Protection des Personnes dans la Recherche Biomédicale (CCPPRB) d’Alsace 1 Strasbourg C.H.R. et U 1, place de L’Hopital F-67091 Strasbourg cedex | Responsible for all French investigators | 4264 3371 4325 2300 3998 3553 4328 3550 2099 3628 4414 3377 2311 1647 4415 1366 1881 | Date of vote: 06.01.2003, further information pending |
| **Hungary** |  |  |  |
| Clinical Pharmacological Ethical Committee of the Scientific Council of Public Health v., Arany János u. 6-8 H-1051 Budapest | responsible for all Hungarian investigators | 3603 4271 3321 | OGYI/191/40//2003 |
| Regionalis Haumán Orbosbiológiai Kutatásetikai Bizottság, Szegedi Tudományegyetem Korányi fasor 8 HU-6720 Szeged | Dr K. Puha | 2523 | Date of vote: 18.02.2003  Reference number:  76-1-4/2003 |
| Regionális Kutatás Etikai Bizottsag Pécsi Orvostudomanyi es Egészségtudományi Központ Szigeti utca 12 HU-7643 Pécs | Dr T. Kecskés | 2969 | No IRB Project # provided, Date of vote: 31.01.2003 |
| Szt Imre Kórhaz Tudományos Kutatásetikai Bizottsága Tétényi ut 12-16 HU-1115 Budapest | Dr K. Major | 3604 | No IRB Project # provided, Date of vote: 08.01.2003 |
| Törökbálinti Tüdögyögyintézet Etikai Bizottsága Munkácsy M u. 70 HU-2045 Törökbálint | Dr Z. Mark | 3404 | No IRB Project # provided, Date of vote: 20.12.2002 |
| **Italy** |  |  | **Date of Meeting = Approval//Protocol no.** |
| Comitato di Bioetica Dell’Azienda Ospedaliera “V. Cervello” di Palermo Via Trabucco 180 I-90146 Palerma | Dr V. Bellia | 4455 | 11.06.2003//2923 |
| Comitato Etico Dell’Azienda Ospedaliera ‚Arcispedale Sant ‚Anna’ Di Ferrara Corso Giovecca 203 I-44100 Ferrara | Dr A. Ciaccia  Dr A. Potena | 2717  4467 | No IRB Project # provided, Date of vote: 27.05.2003  No IRB Project # provided, Date of vote: 27.05.2003 |
| Comitato Etico Dell’Azienda Ospedaliero Universitaria ‘Ospedali Riuniti’ Di Trieste Via Farneto 3 I-34100 Trieste | Dr M. Confalonieri | 5174 | 27.05.2003//145/00 |
| Comitato Etico Provinciale di Modena c/o Azienda Policlinico Via del Pozzo 71 I-41100 Modena | Dr L. Fabbri | 3275 | 11.03.2003//546/CE |
| Comitato Etico Indipendente Dell’Azienda USL Della Citta di Bologna  Via Castiglione 29 I-40124 Bologna | Dr F. Falcone | 4459 | 08.05.2003//522/CE |
| Comitato Etico Dell’Azienda ULSS 15 ‘Alta Padovana’ Via Pietro Cosma 1 I-35012 Camposampiero | Dr G. Idotta | 4462 | 01.04.2003//58618/16.01.02 |
| Comitato Etico per la Sperim. Clin. dei Medicinali Dell’Az. Ospedaliera Universitaria Senese c/o Segreteria di Presidenza della Fac. di Medicina e Chirurgia-C. Didattico le Scotte - Loc. le Scotte I-53100 Siena | Dr M. Rossi | 4471 | No IRB Project # provided, Date of vote: 15.04.2003 |
| Comitato Etico Indipendente Dell’ASL SA/2 Di Salerno Via Nizza 146 I-84124 Salerno | Dr M. Scarpitta | 4473 | 29.05.2003//27181 |
| **Netherlands** |  |  |  |
| Medisch Ethische Toetsingscommissie Martini Ziekenhuis Van Swietenlaan 4 NL-9700 RM Groningen | Dr R. Aalbers | 1869 | 2002-47 |
| Medisch Ethische Toetsingscommissie Postbus 90158 NL-4800 RK Breda | Dr Th. Bantje | 3105 | 612 ml 141 |
| Medisch Ethische Toetsingscommissie Vlietland Ziekenhuis Postbus 1501 NL-3130 GA Vlaardingen | Dr D. Cheung | 3611 | R02-084 |
| Medisch Ethische Toetsingscommissie Chatharina Ziekenhuis Postbus 1350 NL-5602 ZA Eindhoven | Dr J.P. Creemers | 592 | M02/1280 |
| Maxima Medisch Centrum de Medisch Ethische Toetsingscommissie Postbus 7777 NL-5500 MB Veldhoven | Dr D. De Munck | 4280 | 0232 |
| Medisch Ethische Toetsingscommissie Ziekenhuis St. Jansdal Postbus 138 NL-3840 AC Harderwijk | Dr St. Gans | 3327 | BY217/M2-112  No other code listed in correspondence IRB date approval letter 3 December 2002 |
| Elkerliek Ziekenhuis Postbus 98 NL-5700 AB Helmond | Dr W. Pieters | 3103 | No code listed in correspondence IRB date approval letter 30 October 2002 |
| Militair Geneeskundig Facilitair Bedrijf Centraal Militair Hospital Postbus 90 000 NL-3509 AA Utrecht | Dr P. Sips | 3101 | CTWO/2003-02  Kenmerk 3.119-037 |
| Westfries Gasthuis Medisch Ethische Commissie Postbus 600 NL-1620 AR Hoorn | Dr R. Stallaert Dr J. Prins | 4265 | EC151 |
| Medisch Ethische Commissie SMT Postbus 546 NL-7550 AM Hengelo | Dr H. Timmer | 1700 | M02-23 |
| Medisch Centrum Alkmaar METC Noord-Holland Postbus 501 NL-1800 AM Alkmaar | Dr C. de Graaff | 620 | M02-057 |
| Atrium Medisch Centrum Medisch Ethische Commissie Postbus 4446 NL-6401 CX Heerlen | Dr J.A. van Noord | 3102 | 02-P-59 |
| **Poland** |  |  |  |
| Bio-Ethics Committee WIL ul Szaserow 128 PL-00-909 Warszawa | Dr A. Bochenek  Dr Kaczmarek-Czeczotka | 4379  4558 | Resolution No. 7/2003  Resolution No. 7/2003 |
| Bio-Ethics committee a the Regional Medical Chamber ul. Norwida 4 PL-81/434 Gdynia | Dr Gross-Tyrkin  Dr P. Miekus  Dr Piepiorka | 4557  4559  4556 | Resolution No. 187/2003  Resolution No. 185/2003  Resolution No. 186/2003 |
| Academic Bioethics Committee for the Clinical Research at the medical Academy in Lodz 4 Kosciuski Ave PL-90-419 Lodz | Dr I. Grzelewska-Rzymowska  Dr D. Nowak | 4560  4561 | Decision No. RNN/35/03/KE  Decision No. RNN/11/03/KE |
| Bioethics Committee at District Council of Doctors in Bydgoszcz ul. Powstancow Warszawy 11 PL-85-326 Bydgoszcz | Dr Czajkowska-Malinowska | 4562 | Resolution No. 10/2003 |
| Bioethics Committee at Distric Council of Doctors in Lublin Chmielna 4 PL-20-079 Lublin | Dr E. Trebas-Pietras | 4564 | Resolution No. 106/2003/KB |
| **Portugal** |  |  |  |
| Hospital de Sao Joao Servico de Pneumologia Alameda Prof. Dr Hernani Monteiro P-4200-319 Porto | Dr J. de Sousa Almeida | 2010 | IRB Project no.: 58/2002  IRB approval on 06.01.2003; Administration Board approval on 16.01.2003) |
| H. Santa Marta Servico de Pneumologia Rua de Santa Marta P-1150 Lisboa | Dr J. Cardoso | 4281 | Letter received from the Administration Council ref. OF483/CA/MM, 02-12-13  (IRB approval on 21.10.2002; Administration approval on 10.12.02) |
| Hospital Barlavento Algarvio Servico de Pneumologia Sitio do Poco Seco P-8500 Portimao | Dr J. Munha Fernandes | 4282 | No IRB Project or registration number; Letter of approval received from the Hospital dated 003/01/14 (received on 17.01.2003) |
| H. Fernando do Fonseca  Servico de Pneumologia - IC 19 P-2700 Amadora | Dr M. Rodrigues | 3962 | No IRB Project or registration number; IRB approval on 07.01.2003 |
| **Russia** |  |  |  |
| Ethics Committee at the Federal Agency of Control over Drug Quality, Efficacy and Safety Petrovskii Bulvar 8, Build 1 RU-103051 Moscow | responsible for all Russian investigators | 4589 4591 4595 4581 4594 4588 4593 | Extract of Minutes No. 35 Meeting of the Ethics Committee at the Federal Agency of Control over Drugs Quality, Efficacy and Safety  Case No. 1386 |
| Ethics Committee at Russian State Medical University Ostrovityanova, str. 1 RU-117997 Moscow | Dr Y. Belousov Dr B. Bart | 4592 4590 | Extract from RSMU EC Meeting Minutes No. 27 of 23 June 2003 |
| **South Africa** |  |  |  |
| Pharma Ethics (PTY.) Ltd. 123 Amcor Road Lyttelton Manor RSA-0157 | On behalf of all South African investigators | 4022 4288 4113 4283 4286 4285 3782 1459 4287 4284 | Date of vote: 06.11.2002, further information pending |
| UCT Lung Istitute Research Ethics Committee Faculty of Health Sciences University of Cape Town George Street Mowbray RSA-7925 Cape Town | Dr E. Bateman | 3397 | Date of vote: 23.01.2003, further information pending |
| The University of Free State Faculty of Health Sciences Nelson Mandela Road RSA-9300 Blomfontein | Dr M. Prins | 3399 | Date of vote: 22.10.2002, further information pending |
| Tygerberg Ethics Committee Pharmaceutical Trial Advisory Committee Medical School Tygerberg Campus SA-7505 Tygerberg | Dr E. Irusen | 655 | Date of vote: 26.03.2003, further information pending |
| **Spain** |  |  |  |
| Ethics Committee of Hospital Universitario Puerta del Mar Avda. Ana de Viya, 21 E-11009 Cadiz | Dr A. Armedillo | 4292 | No IRB Project # provided, Date of vote: 04.12.2002 |
| Ethics Committee of Hospital Dr Negrin Barranco de la Ballena, s/n E-35019 Las Palmas de Gran Canaria | Dr P. Cabrera | 4193 | No IRB Project # provided, Date of vote: 12.12.2002 |
| Regional Ethics Committee of Madrid C/ Aduana, 29 E-28013 Madrid | Dr N. Abad Santamaría Dr P. de Lucas Dr J. Echave-Sustaeta Dr B. Steen | 3792 1280 3793 4192 | No IRB Project # provided, Date of vote: 29.01.2003 |
| Ethics Committee of Hospital Universitario Infanta Cristina Ava. de Elvas, s/n E-06080 Badajoz | Dr F. Fuentes | 3976 | No IRB Project # provided, Date of vote: 23.10.2002 |
| Ethics Committee of Hospital de Cruces Hospital de Cruces Plaza de Cruces, s/n E-48903 Barakaldo | Dr J. Galdiz | 1282 | No IRB Project # provided, Date of vote: 04.12.2002 |
| Ethics Committee of Hospital General Universitario de Alicante Maestro Alonso, 109 E-03010 Alicante | Dr E. Llorca Martinez | 3619 | No IRB Project # provided, Date of vote: 07.05.2003 |
| Ethics Committee of Hospital Miguel Servet Paseo Isabel La Católica, 1-3 E-50009 Zaragoza | Dr J. Marin-Trigo | 4290 | No IRB Project # provided, Date of vote: 21.01.2003 |
| Regional Ethics Committee of Galicia San Lazaro, s/n E-17703 Santiago de Compostela | Dr J. Rodríguez Suarez  Dr L. Valdes | 3153 4291 | No IRB Project # provided, Date of vote: 20.11.2002 |
| Ethics Committee of Hospital General Universitario de Elche Cami de L’Almazara, 11 E-03203 Elche (Alicante) | Dr C. Shum Funk | 2199 | No IRB Project # provided, Date of vote: 12.12.2002 |
| Ethics Committee of Hospital Arnau de Vilanova San Clemente, 12 E-46015 Valencia | Dr F. Sanchez Toril | 1265 | No IRB Project # provided, Date of vote: 18.12.2002 |
| Ethics Committee of Hospital Clinic i Provincial,  Unidad de Ensayos Clinicos Hospital Clinic i Provincial C/Villarroel, 170 E-08036 Barcelona | Dr A. Torres Marti | 2606 | No IRB Project # provided, Date of vote: 06.11.2002 |
| Regional Ethics Committee of Galicia San Lázaro, s/n E-17703 Santiago de Compostela | Dr H. Verea | 1288 | No IRB Project # provided, Date of vote: 20.11.2002 |
| **Switzerland** |  |  |  |
| Ethikkommission beider Basel (EKBB) Hebelstr. 53 CH-4056 Basel | Dr A. Breitenbücher  Prof. Dr M. Tamm | 4800  231 | No. 215/02 |
| Kantonale Ethikkommission Bern (KEK) Postfach 56 CH-3010 Bern | Dr W. Bauer Dr J. Leuppi  Dr H.-U. Bettschen | 4307  3092 | No. 189/02 |
| Kantonale Ethikkommission Gesundheitsdirektion Kt. Zürich Haldenbachstr. 12 CH-8033 Zürich | Dr U. Honegger  Dr J. Barandun  Dr M. Häcki  Dr E. Imhof | 4305  4303  4304  157 | No. 01/03 |
| Comité Intercantonal D’Ethique Jura, Fribourg, Neuchatel  Maison de santé de Préfargier, Direction medicale  CH-2074 Marin | Dr J.-P. Ketterer | 4306 | No. 25/2002 |
| **United Kingdom** |  |  |  |
| West Essex Local Research Ethics Committee c/o The Princess Alexandra Hospital NHS Trust Terminus House The High UK-Harlow CM20 1XA | Dr G. Ambepitiya | 4294 | Date of vote: 20.12.2002, further information pending |
| North Sheffield Local Research Ethics Committee Northern General Hospital Herries Road UK-Sheffield S5 7AU | Dr P. Anderson | 1855 | Date of vote: 10.12.2002, further information pending |
| South Sefton Local Research Ethics Committee The Walton Centre for Neurology & Neurosurgery C/o Clinical Trials Unit Lower Lane Fazakerley UK-Liverpool L9 7LJ | Prof. Dr P. Calverley | 4295 | Date of vote: 23.12.2002, further information pending |
| Sunderland Local Ethics Research Committee Durham Road UK-Sunderland SR3 4AF | Dr G. McBride | 4298 | Date of vote: 27.01.2003, further information pending |
| Brighton & Hove PCT Vantage Point, 6^th^ Floor New England Road UK-Brighton BN1 4GW | Dr D. Dutchmann | 2235 | Date of vote: 17.12.2002, further information pending |
| Cambridge Local Research Ethics Committee Box 148 Addenbrooke’s NHS Trust Hills Road UK-Cambridge CB2 2QQ | Dr A. George | 4073 | Date of vote: 12.12.2002, further information pending |
| Newcastle & North Tyneside Local Research Ethics Committee University of Newcastle Framlington Place UK-Newcastle-upon-Tyne NE2 4HH | Dr G. Gibson  Dr S. Stenton | 4297  4302 | Date of vote: 18.02.2003, further information pending |
| Bath Local Research Ethics Committee Forbes Fraser Building Bath Royal United Hospital Combe Park UK-Bath BA1 3NG | Dr J. Hamling | 1987 | Date of vote: 20.01.2003, further information pending |
| Ethics Committee Adelaide & Meath Hospital Tallaght IR-Dublin 24 | Dr P.F. Lane | 4299 | Date of vote: 13.12.2002, further information pending |
| Greater Glasgow Community/Primary Local Research Ethics Committee Trust Head Quarters Gart Navel Royal Hospital 1055 Great Western Road UK-Glasgow G12 0XH | Dr J. Langan  Dr C. McKinnon | 4300  3948 | Date of vote: 15.01.2003, further information pending |
| South Manchester Local Research Ethics Committee Manchester Health Authority Gateway House Piccadilly South UK-Manchester M60 7LP | Dr St. Langley | 4074 | Date of vote: 16.01.2003, further information pending |
| Southmead Local Research Ethics Committee Clinical Governance Directorate Beaufort House Southmead Hospital Westbury-on-Trym UK-Bristol BS10 5NB | Dr A. Millar | 4301 | Date of vote: 23.12.2002, further information pending |
| Bedfordshire & Hertfordshire Strategic Health Authority Local Research Ethics Committee Tonman House 63-67 Victoria Street UK-St Albans AL1 3ER | Dr N. Savani | 3622 | Date of vote: 15.01.2003, further information pending |

**Appendix 3**

**Adverse events**

During treatment, adverse events were reported by 1081 (81.5%) patients in the roflumilast group and 1089 (80.1%) patients in the placebo group (Table 3), of which 285 (21.5%) patients in the roflumilast group and 113 (8.3%) in the placebo group had events that were assessed to be likely or definitely related to the study drug. The most frequently reported adverse event was exacerbation of COPD (roflumilast, 42.9% [n=569/1327]; placebo, 48.0% [n=652/1359]), whereas most of the causally related adverse events in the roflumilast group affected the gastrointestinal tract, most commonly diarrhea (roflumilast, 12.1%; placebo, 2.9%) followed by nausea (roflumilast, 6.0%; placebo, 1.5%) and weight loss (roflumilast, 7.5%; placebo, 2.8%). In most cases adverse events resolved with continued treatment. The incidence of serious adverse events was similar in the two groups (roflumilast, 19.8%; placebo, 19.4%); the most common was COPD exacerbation (roflumilast, 10%; placebo, 10.1%) followed by pneumonia (roflumilast, 1.8%; placebo 1.9%). This pattern of adverse events was similar in the subgroups. For the groups with chronic bronchitis, in whom a greater benefit of roflumilast was observed on exacerbations and SGRQ, there were numerically fewer adverse events and fewer serious adverse events. This reduction was greatest for diarrhea (7.1% vs. 18.5%), nausea (4.4% vs. 8%) and weight loss (6.1% vs. 11.9%), although these were higher with roflumilast than with placebo. Of the most common adverse events in roflumilast-treated patients, only pneumonia was more common among the chronic bronchitis patients (3.5% vs. 1.7%); however, pneumonia was more common in the placebo subjects both with and without chronic bronchitis. There were also more adverse events in the subgroup of patients receiving concomitant ICS, but the relative rates between roflumilast and placebo were comparable for patients with and without ICS treatment (Table 3). Importantly, the subset analysis did not identify any group of subjects that appeared to be at higher risk of adverse events from roflumilast.

**Table S1 Lung function results summary table (change in lung function variable after 52 Weeks compared with b**aseline)

| **Characteristic** | **Roflumilast** | | **Placebo** | | **Roflumilast – placebo difference (95% CI)** | **p value** |
| --- | --- | --- | --- | --- | --- | --- |
| **Pre-bronchodilator FEV_1_, mL (SE)** | **n** | **Change from baseline*** | **n** | **Change from baseline*** |  |  |
| M2-111 | 545 | 30 (7.8) | 596 | –12 (7.3) | 42 (22, 61) | < 0.0001 |
| M2-112 | 737 | 49 (9.2) | 741 | –8 (9.0) | 57 (37, 77) | < 0.0001 |
| Pooled results |  |  |  |  |  |  |
| Overall | 1282 | 41 (6.1) | 1337 | –10 (5.8) | 51 (37, 65) | < 0.0001 |
| Sex:  Female  Male | 350  932 | 43 (9.0)  62 (6.6) | 381  956 | 8 (8.4)  5 (6.4) | 35 (11, 59)  57 (40, 75) | 0.0045  < 0.0001 |
| Smoking status:  Current  Former smoker | 512  770 | 44 (10.3)  44 (6.6) | 523  814 | 1 (9.9)  –13 (6.3) | 43 (16, 70)  57 (41, 73) | 0.0017  < 0.0001 |
| Concomitant treatment:  ICS  No ICS | 776  506 | 42 (6.9)  46 (9.9) | 803  534 | –11 (6.6)  –3 (9.6) | 53 (36, 70)  49 (23, 74) | < 0.0001  0.0002 |
| Concomitant treatment:  Short-acting anticholinergics  No short-acting anticholinergics | 759  523 | 38 (7.0)  59 (9.7) | 806  531 | –4 (6.7)  –6 (9.5) | 42 (25, 59)  65 (39, 90) | < 0.0001  < 0.0001 |
| COPD severity:  Very severe  Severe | 317  835 | 51 (9.6)  48 (7.4) | 337  896 | 23 (9.3)  –15 (6.9) | 29 (7, 50)  63 (44, 81) | 0.0086  < 0.0001 |
| COPD history:  Emphysema | 343 | 54 (9.7) | 406 | –6 (8.9) | 60 (37, 84) | < 0.0001 |
| Chronic bronchitis ± emphysema | 800 | 45 (7.3) | 839 | –2 (7.0) | 46 (28, 65) | < 0.0001 |
| Chronic bronchitis ± emphysema:  ICS  No ICS | 478  322 | 48 (8.9)  34 (12.3) | 491  348 | –7 (8.6)  0 (11.9) | 55 (33, 77)  34 (2, 65) | < 0.0001  0.0357 |
| Cough score at Week 0: |  |  |  |  |  |  |
| ≥ 1 (average/day) | 863 | 60 (7.2) | 924 | –3 (6.8) | 63 (44, 81) | < 0.0001 |
| < 1 (average/day) | 385 | 26 (10.0) | 379 | –9 (10.2) | 35 (11, 58) | 0.0046 |
| Sputum score at Week 0 |  |  |  |  |  |  |
| ≥ 1 (average/day) | 799 | 53 (7.2) | 848 | –6 (6.8) | 58 (41, 76) | < 0.0001 |
| < 1 (average/day) | 445 | 46 (10.1) | 453 | 3 (10.2) | 42 (17, 68) | 0.0011 |
| Study completion status |  |  |  |  |  |  |
| Completers | 890 | 45 (6.6) | 1008 | –6 (6.2) | 51 (35, 68) | <0.0001 |
| Non-completers | 392 | 52 (13.7) | 329 | 10 (13.2) | 42 (6, 79) | 0.0218 |
| **Post-bronchodilator FEV_1_, mL (SE)** | | | | | | |
| M2-111 | 543 | 26 (8.1) | 592 | –16 (7.5) | 42 (22, 63) | < 0.0001 |
| M2-112 | 732 | 56 (9.3) | 742 | –4 (9.2) | 60 (40, 81) | < 0.0001 |
| Pooled results |  |  |  |  |  |  |
| Overall | 1275 | 43 (6.2) | 1334 | –10 (6.0) | 53 (39, 68) | < 0.0001 |
| Sex  Female  Male | 346  929 | 36 (9.3)  62 (6.7) | 379  955 | 14 (8.7)  –4 (6.6) | 21 (–4, 46)  65 (47, 83) | 0.0934  < 0.0001 |
| Smoking status:  Current smoker  Former smoker | 510  765 | 42 (10.5)  46 (6.7) | 518  816 | –6 (10.1)  –12 (6.5) | 47 (20, 75)  58 (42, 75) | 0.0007  < 0.0001 |
| Concomitant treatment:  ICS  No ICS | 770  505 | 34 (7.1)  54 (10.0) | 801  533 | –19 (6.8)  2 (9.8) | 54 (36, 71)  53 (27, 78) | < 0.0001  < 0.0001 |
| Concomitant treatment:  Short-acting anticholinergics  No short-acting anticholinergics | 755  520 | 32 (7.3)  66 (9.7) | 805  529 | –10 (6.9)  –4 (9.5) | 42 (25, 60)  69 (44, 94) | < 0.0001  < 0.0001 |
| COPD severity:  Very severe  Severe | 313  834 | 72 (10.9)  56 (7.5) | 339  892 | 41 (10.4)  –13 (7.0) | 31 (7, 55)  69 (51, 88) | 0.0124  < 0.0001 |
| COPD history  Emphysema | 342 | 48 (9.8) | 404 | –11 (9.1) | 59 (35, 83) | < 0.0001 |
| Chronic bronchitis ± emphysema | 799 | 48 (7.5) | 839 | –3 (7.3) | 51 (31, 7.0) | < 0.0001 |
| Chronic bronchitis ± emphysema:  ICS  No ICS | 478  321 | 44 (9.3)  44 (12.5) | 491  348 | –16 (9.0)  8 (12.1) | 60 (36, 83)  36 (4, 68) | < 0.0001  0.0265 |
| Cough score at Week 0 |  |  |  |  |  |  |
| ≥ 1 (average/day) | 860 | 60 (7.4) | 923 | –9 (6.9) | 69 (5.1, 88) | < 0.0001 |
| < 1 (average/day) | 381 | 16 (10.0) | 377 | –11 (10.2) | 27 (3, 51) | 0.0252 |
| Sputum score at Week 0 |  |  |  |  |  |  |
| ≥ 1 (average/day) | 796 | 52 (7.5) | 847 | –8 (7.0) | 61 (42, 79) | < 0.001 |
| < 1 (average/day) | 441 | 37 (10.0) | 451 | –8 (10.0) | 45 (20, 70) | 0.0004 |
| Study completion status |  |  |  |  |  |  |
| Completers | 892 | 50 (6.8) | 1008 | –8 (6.4) | 58 (42, 75) | <0.0001 |
| Non-completers | 383 | 26 (13.4) | 326 | –3 (13.2) | 29 (–7, 65) | 0.114 |

* Repeated measures analysis

***Table S2 St George’s Respiratory Questionnaire (SGRQ) total score: change after 52 Weeks compared with baseline***

| **Patient subset**  **(M2-111 + M2-112 pool)** | **Roflumilast** | | **Placebo** | | **Roflumilast – placebo difference (95% CI)** | **2-sided**  **p value** |
| --- | --- | --- | --- | --- | --- | --- |
|  | **n** | **Change from baseline*** | **n** | **Change from baseline*** |  |  |
| M2-111 | 340 | –2.749 | 410 | 0.006 | –2.755 (–4.420, –1.089) | 0.0012 |
| M2-112 | 530 | –3.754 | 584 | –3.186 | –0.568 (–2.028, 0.892) | 0.4454 |
| Pooled results |  |  |  |  |  |  |
| Overall | 1087 | –2.253 | 1234 | –1.655 | –0.599 (–1.394, 0.196) | 0.1396 |
| Sex |  |  |  |  |  |  |
| Female | 273 | –2.821 | 346 | –2.276 | –0.544 (–2.084, 0.995) | 0.4879 |
| Male | 814 | –2.066 | 888 | –1.280 | –0.785 (–1.726, 0.155) | 0.1016 |
| Smoking status |  |  |  |  |  |  |
| Current | 442 | –3.179 | 469 | –2.125 | –1.054 (–2.348, 0.241) | 0.1105 |
| Former smoker | 645 | –1.474 | 765 | –0.964 | –0.510 (–1.530, 0.510) | 0.3271 |
| Concomitant treatment: |  |  |  |  |  |  |
| ICS | 655 | –2.062 | 746 | –1.264 | –0.798 (–1.832, 0.237) | 0.1305 |
| No ICS | 432 | –2.293 | 488 | –1.830 | –0.463 (–1.736, 0.809) | 0.4753 |
| Concomitant treatment: |  |  |  |  |  |  |
| Short-acting anticholinergics | 634 | –1.964 | 749 | –1.376 | –0.588 (–1.592, 0.416) | 0.2510 |
| No short-acting anticholinergics | 453 | –2.461 | 485 | –1.645 | –0.815 (–2.135, 0.504) | 0.2256 |
| COPD severity |  |  |  |  |  |  |
| Very severe | 252 | –1.203 | 305 | –0.825 | –0.378 (–2.038, 1.282) | 0.6549 |
| Severe | 717 | –2.038 | 829 | –1.370 | –0.669 (–1.658, 0.320) | 0.1850 |
| COPD history |  |  |  |  |  |  |
| Emphysema | 298 | –1.580 | 377 | –1.682 | 0.102 (–1.478, 1.683) | 0.8988 |
| Chronic bronchitis ± emphysema | 734 | –2.658 | 803 | –1.585 | –1.073 (–2.022, –0.125) | 0.0265 |
| Chronic bronchitis ± emphysema: |  |  |  |  |  |  |
| ICS | 438 | –2.744 | 472 | –1.462 | –1.282 (–2.504, –0.060) | 0.0397 |
| No ICS | 296 | –2.457 | 331 | –1.643 | –0.814 (–2.328, 0.700) | 0.2916 |
| Cough score at Week 0: |  |  |  |  |  |  |
| ≥ 1 (average/day) | 743 | –2.739 | 855 | –2.075 | –0.663 (–1.645, 0.318) | 0.1851 |
| < 1 (average/day) | 315 | –1.005 | 347 | –0.040 | –0.965 (–2.385, 0.455) | 0.1825 |
| Sputum score at Week 0 |  |  |  |  |  |  |
| ≥ 1 (average/day) | 685 | –2.649 | 782 | –1.843 | –0.806 (–1.835, 0.224) | 0.1250 |
| < 1 (average/day) | 369 | –1.571 | 416 | –1.171 | –0.401 (–1.692, 0.891) | 0.5427 |
| Study completion status |  |  |  |  |  |  |
| Completer | 882 | –2.743 | 1002 | –1.970 | –0.772 (–1.632, 0.088) | 0.0784 |
| Non-completer | 205 | –0.690 | 232 | 3.286 | –3.977 (–8.033, 0.079) | 0.0546 |

* Repeated measures analysis

**Figure S1 Trial profile of M2-111** Percentages are based on the number of randomized patients in a treatment group.
