# Supplementary material for: Reduction of exacerbations by the PDE4 inhibitor roflumilast - the importance of defining different subsets of patients with COPD
Source: Respir Res. 2011 Jan 27;12(1):18. doi: 10.1186/1465-9921-12-18 (PMC3040135; doi:10.1186/1465-9921-12-18)
Supplement: Additional file 2 — List of investigators for Studies M2-111 and M2-112. M2-111 investigators; M2-112 investigators. [file 1465-9921-12-18-S2.DOC]

# Additional File 2

# Exacerbation reduction by roflumilast – importance of defining different subsets of patients with COPD

Stephen I Rennard^1^, Peter MA Calverley^2^, Udo M Goehring^3^, Dirk Bredenbröker^3^, Fernando J Martinez^4^

^1^Nebraska Medical Center, Omaha, USA, ^2^University Hospital Aintree, Liverpool, UK, ^3^Nycomed GmbH, Konstanz, Germany, ^4^University of Michigan Medical Center, Ann Arbor, USA

**List of investigators for Studies M2-111 and M2-112**

**M2-111 Investigators**

*Canada*

Jean Bourbeau, Montreal Chest Clinic, Montreal, PQ; Pierre Larivée, Universite de Sherbrooke, Sherbrooke, PQ; Andrew McIvor, New Halifax Infirmary, Halifax, Nova Scotia; Kenneth R. Chapman, Toronto, ON; Jacques, Lenis, Invascor Clincal Research, Longueuil, PQ; Michel Rouleau, CHA-Hospital Saint Sacrement, Quebec, QC; Anthony Dowell, Clinical Research Consultant Group, Montreal, PQ; Lawrence Homik, Concordia Hospital, Winnipeg, MB; Andrew Cave, University of Alberta, Edmonton; Sam Henein, New Market, ON; Victor Hoffstein, St. Michael's Hospital, Toronto, ON; Robert Kennedy, Professional Corporate, Calgary; Arun Nayar, Nayar Arun, Saskatoon, SK; Victoria Chan, Digestive Health Clinic, Richmond Hill, ON; Gary Kivinen, Northern Centre for Biotech, Sudbury, ON; Brian Carlson, North Road Medical Centre, Coquitlam, BC; Reena Dhatt, Neureka Research Corporation, Sudbury, ON.

*Germany*

Heiner Steffen, Landsberg/Lech; Dieter, Rost, Augsburg; Hans-Hermann Ponitz, Berlin; Hermann A. Trauth, Marburg; Joachim Pettenkofer, Berlin; Jörg Eller, Berlin; Vera Grimm-Sachs, Bruchsal; Thomas Ginko, Bonn; Umberto Gehling, Schwetzingen; Gert Kunkel, Allergie- und Asthmazentrum Westend, Berlin; Andreas Piecyk, Pneumologisches Forschungsinstitut GmbH, Magdeburg; Norbert Reinholz, Augsburg; Janos Weoeres, München; Wolfgang Huebner, Dillingen an der Donau; Thomas Dapper, Saarbruecken.

*France*

Christian Sevette, Centre Hospitalier, Perpignan; Pierre Zuck, Hôpital du Bon Secours, Metz; Henri Kafé, Cabinet de Pneumologie, Saint-Quentin; Roger Escamilla, Hôpital Purpan, Toulouse; Jacques Dupouy, Cabinet de Pneumologie, Nimes; Herve Pegliasco, Hopital Ambroise Pare, Marseille; Bernard Bugnas, Cabinet de Pneumologie, Nice; Herve Jullian, Centre Hospitalier General, Martigues; Laurent Fouquert, Cabinet de Pneumologie, Grasse; Lucien Bernabeu, Centre Hospitalier de Chauny, Chauny; Christophe Verkindre, Centre Hospitalier de Béthune, Beuvry; Jean-Francois Cordier, Hópital Cardiovasculaire et Pneumologique, Lyon; Catherine LAmblin, CH Lens, Lens; Stéphane Beaujot, Cambrai.

*Poland*

Bozena Klimkiewicz, Centrum Leczenia Chorob Cywilizacyjnych, Warsaw; Marek Piepiorka, Szpital Specjalistyczny-Oddzial, Wejherowo; Ewa Gross-Tyrkin, General Practice "Non Nocere", Gdansk; Pawel Miekus, Szpital Miejski w Gdyni-Oddzial, Gdynia; Iwona Grzelewska-Rzymowska, Uniwerytet Medyczny w Lodzi Kilnika, Lodz; Dariusz Nowak, Osrodek Diagnostyki i Leczenia, Lodz; Ewa Trebas-Pietras, Wojewodzki Szpital im. Kardynala, Lublin; Jan Cieslicki, Wojewodzki Szpital Chorob Pluc, Wodzislaw Sl.; Krystyna Folcik, Samodzielny Specjalistyczny ZOZ w Rudka, Mrozy; Maria Bizunowicz-Artecka, Poradnia Gruzlicy I Chorob Pluc SPZOZ, Warsaw.

*South Africa*

Gerhard Ras, Muelmed Medical Centre 506, Arcadia, Pretoria; Analene Nel, Clinical Research Centre, Paarl; Eric Bateman, UCT Lung Institute, Mowbray, Cape Town

Michelle V. Middle, Clinical Trials (SACT), George; Mohamed Sabeer Abdool-Gaffar, Kingsway Clinical Trial Centre, Durban, Amanzimtoti; Sanet Visser, Pretoria; Gysbert Charl Liebenberg, Durbanville Medic-Clinic, Durbanville / Cape Town; Johann J. Viljoen, Genclin Corporation, Bloemfontein; EM Irusen, Respiratory Research Dep. of Int. Med., Cape Town, Tygerberg; Alwyn Foden, Panorama Medical Centre, Panorama /Cape Town; JG Killian, Wilmed Medical Research Projects, Pretoria; LP Krige, Dr. LP Krige, Port Elizabeth; JA Venter, Garsfontein / Pretoria; W Labuschagne, Intercare Health Centre, Pretoria; JA O'Brien, Christiaan Barnard Memorial Hospital, Cape Town; Jacob Jansen, Mediclinic Vergelegen, Cape Town; Dorelle L Kirsten, Jacaranda Hospital, Pretoria.

*USA*

E. Neil Schachter, The Mount Sinai Medical Center, New York; Melvin Morganroth, The Oregan Clinica, Portland; Antonio Anzueto, Audie Murphy VA Medical Center, San Antonio; Charles Kistler, Remington-Davis Inc, Columbus; Dennis E. Doherty, University of Kentucky Medical Center, Lexington; Ware Kuschner, VA Medical Center--Palo Alto, Palo Alto; Daniel Lorch, Pulmonary Assoc. of Brandon, Brandon; Neil Horning, Chest Infectious Disease and CC Assoc., Des Moines; Eric J. Schenkel, Valley Clinical Research Center, Easton; David I. Bernstein, Berstein Clin. Research Center, Cincinnati, OH; Kalpalatha Guntupalli, Baylor College of Medicine, Houston; Harold S. Nelson, National Jewish Medical+Research Center, Denver; Harold B. Kaiser, Clinical Research Institute, Minneapolis; James Wolfe, Allergy & Asthma Associates of Santa Cla, San Jose; Phillip Korenblat, The Clinical Research Center, LLC, St. Louis; Bruce G. Rankin, University Clinical Research, Deland; Robert Benkert, Western States Clinical Research, Inc., Arvada; Gilbert Salazar, Sun Research Institute, San Antonio; Suzanne Weakley, Oak Medical Arts Complex, Houston; John A. Winder, Toledo Center for Clinical Research, Sylvania; Arthur C. DeGraff, Physicians Research Center, Inc., Hartford; Thomas M. Siler, Midwest Chest Consultants, PC, St. Charles; Wayne Sinclair, Montana Medical Research, Missoula; Adam Wanner, University of Miami School of Medicine, Miami; Edward Diamond, Alexian Brothers Center for Clinical Re., Elk Grove Village; Randall Bell, Diagnostics Research Group, San Antonio; Timothy J. Craig, M.S. Hershey Medical Center, Hershey; Jonathan Ilowite, Winthrop Hospital, Mineola; Michael C. Kallay, Highland Hospital, Rochester; Edward M. Kerwin, Clin. Research Inst. of Southern Oregon, Medford; Robert Sussman, Pulmonary Allergy Associates, Springfield; Steven A. Sahn, Medical University of South Carolina, Charleston; Mary E. Strek, University of Chicago, Chicago; Mark C. Liu, John Hopkins Asthma & Allergy Center, Baltimore; Jay B. Enden, Pulmonary Medical Research of NYLC, Bay Shore, NY; Vicki Greiff, Alta Clinical Research, Tucson, AZ; James Nishio, Pulmonary Medical Associates, Sacramento; Amy Silverthorn, Amy Silverthorn, MD, Phoenix, AZ; Simon Babazadeh, Crest Clinical Trials, Anaheim; James Baker, Allergy, Asthma and Dermatology, Lake Oswego; Adnan Dahdul, FutureCare Studies, Springfield; Edward E. Lisberg, Asthma & Allergy Center of Chicago, River Forest; Irwin Spirn, Delaware Valley Clinical Research, Cherry Hill; Leonard J. Dunn, Clinical Research of West Florida, Clearwater; Robert Wolfe, Office of Robert N. Wolfe, MD, Los Angeles; Barry Streit, Medical Pulmonary Associates, Tamarac; Gordon Raphael, Raphael, Bethesda; Sandra K. Willsie, Kansas City University, Kansas City; David Hill, Waterbury Pulmonary Associates, Waterbury, CT; E. Schelbar, Healthcare Research Consultants, LLC, Tulsa; Anjuli Nayak, Sneeze, Wheeze & Itch Associates, LLC, Normal; Paul Chervinsky, Northeast Medical Research Associates, North Dartmouth; Stephen G. Basheda, South Hills Pulmonary Associates, Pittsburgh; B. Lauren Charous, Advanced Healthcare, Milwaukee; Marc F. Jacobs, Bend Memorial Clinical Research Dep., Bend; Thomas D. Kaelin, Jr., Lowcountry Lung & Critical Care, PA, Charleston, SC; Delbert Meyer, Carmichael; Jackson M. Rhudy, Optimum Clinical Research, Salt Lake City; John Scott Sibille, Sunset Medical Research, Sunset; W. Travis Ellison, Radiant Research- Greer, Greer; Richard Kahn, Central Maine Pulmonary Associates, Auburn; Parimal Parikh, Best Clinical Trials, LLC, New Orleans; Javier Quesada, West Coast Clinical Trials, Long Beach; Roger A. Abrahams, Morgantown Pulmonary Associates, Morgantown; Nizar Daboul, Clinical Research Source Inc., Perrysburg; William E. Berger, Southern California Institute, Mission Viejo; James Lampasso, Buffalo Cardiology and Pulmonary Assoc, Williamsville; T. Shull Lemire, Northwest Physicians Research Network, Missoula; Albert M. Baker, Lynchburg Pulmon. Associates, Inc., Lynchburg; Frank N. Cole, Fayette Medical Clinic, Fayetteville; Michael E. Manning, Allergy and Immunology Associates, Scottsdale; Philip Marcus, Nassau Chest Physicians, Great Neck; Peter B. Platzer, Holston Medical Group Clinical Research, Kingsport; Lawrence Repsher, Pulmonary Research Consultants, Wheat Ridge; Anthony R. Rooklin, Asthma and Allergy Research Association, Upland; Paul Sachs, Pulmonary Association of Stamford, Stamford; Ray Tidman, River Birch Research, Blue Ridge; James Hoyt, Northern Colorado Pulmonary Consultants, Fort Collins; Stephen M. Kreitzer, Dr. Stephen Kreitzer, Tampa; Rana Rab-Hasan, AIM Research, Atlanta; Omer Abdullah, Hunter Holmes McGuire VA Clinic, Fredericksburg; Tahir Ahmed, Mount Sinai Med. Center-Pulmon. Div., Miami Beach; C. Lynn Anderson, Bay Pines VA Medical Center, Bay Pines; Bill Bailey, University of Alabama at Birmingham, Birmingham; Leonard Bielory, UMDNJ, Newark; Eugene Bleecker, Cloverdale Research Facility, Winston Salem; David Cardona, Pharmaseek, Marietta; J. Allen D. Cooper, Jr., Birmingham VA Medical Center, Birmingham; Gerald S. Davis, Vermont Lung Center, Colchester; James Donohue, Univ of North Carolina at Chapel Hill, Chapel Hill; Joel D. Epstein, Southern California Clinical Trials, Lakewood; David L. Fried, Omega Medical Research, Warwick; Ashok M. Fulambarker, North Chicago VA Medical Center, North Chicago; Ronald M. Gilman, Safe Harbor Clinical Research, East Providence; Mark H. Gotfried, Pulmonary Associates, Glendale; Nicholas Gross, Hines VA Hospital, Hines; Michael P. Habib, Southern Arizona VA Health Care System, Tucson; Jeffrey R. Hammersley, Medical University of Ohio, Toledo; Frederick Charles Hiller, ll, University of Arkansas for Med. Sciences, Little Rock; Stanley R. Horner, Allergy, Asthma, Immunology Services, Jefferson City; Frederick W. Kahn, Montana Health Research Institute, Billings; Jill Karpel, Northshore- LIJ Health System, New Hyde Park; Steven Kelsen, Temple University Hospital, Philadelphia; David W. Koh, Illinois Heart & Lung Associates, SC, Bloomington; Peter Krumpe, VA SNHCS-Reno, Reno; Robert S. Larimer, Amulet, LLC, New Orleans; David C. Levin, VA Medical Center, Oklahoma City; Thomas B. O'Barr, nTouch Research Corporation, Marietta; Michael Littner, VA Medical Center-Sepulveda, Sepulveda; Donald A. Mahler, Dartmouth-Hitchcock Medical Center, Lebanon; C. Kees Mahutte, Long Beach VA Medical Center, Long Beach; Thomas J. Martin, VA Medical Clinic, Salem; John Murray, Vanderbilt ASAP, Nashville; Vandana Patel, MedSource, Inc., Chesapeake; Thomas J. Ferro, McGuire VA Medical Center, Richmond; James L. Pearle, California Research Medical Group, Inc., Fullerton; Craig A. Piquette, Veterans Affairs Medical Center, Omaha; Jeffrey Rehm, Pulmonary Associates of Fredericksburg,, Fredericksburg; Stephen Rennard, University of Nebraska Medical Center, Omaha; Kathryn L. Rice, VA Medical Center, Minneapolis; Andrew Ries, UCSD Medical Center, San Diego; Amir Sharafkhaneh, Houston VA Medical Center, Houston; Jitendra Singh, nTouch Research Corporation, Decatur; Selwyn Spangenthal, American Health Research, Charlotte; Donald Tashkin, UCLA Medical Center, Los Angeles; Janine Vintch, Harbor UCLA Research & Education Institute, Torrance; Tony E. Warren, Harbin Clinic, Rome; Paul Weinberg, Gwinett Pulmonary Group, Lawrenceville; David C. Willms, Sharp Memorial Hospital, San Diego; Richard L. ZuWallack, St. Francis Hospital & Medical Center, Hartford; Todd Horiuchi, Sarasota Memorial Hospital, Sarasota; Stephen Krinzman, University of Massachusetts Memorial Med, Worcester; Andrew J. Pedinoff, Princeton Center for Clinical Research, Skillman; Kevin Kovitz, Tulane University, New Orleans; John Thiele, Best Clinical Trials, New Orleans; Curtis Mello, Infinity Medical Research, Inc., North Dartmouth; Jonathan Fine, Norwalk Hospital, Norwalk; Keith Wilkens, South Hill Family Medicine, Spokane; Stephanie Eaton, Northside Respiratory Care, Atlanta; John Cohn, Asthma, Allergy & Pulmonology Associates, Philadelphia; Mitchell G. Kaye, Minnesota Lung Center, Minneapolis; Douglas Puryear, Pulmonary Associates of Richmond, Inc., Richmond; John Jayne, Asthma & Pulmonary Diagnostic Associates, Absecon; Michael Alter, Minnesota Lung Center, Edina; Henry Covelli, Pulmonary and Internal Medicine, Coeur d'Alene; Brian Snyder, Southgate Medical Group, West Seneca; Steven Elliot, Medisphere Medical Research Center, LLC, Evansville; Gershwin Blyden, TuKoi Institute for Clinical Research, Miami; Anarshiravan Hami, Chest & Critical Care Consultants, Anaheim; Gregory Peterson, Diagnostic & Critical Care Medicine, PC, Des Moines.

**M2-112 Investigators**

*Austria*

Hartmut Zwick, Wien; Johann Grillenberger, Perg; Gerlind Holub, Steyr; Wolfgang Pohl, Gänserndorf; Mahmud Sweilem, Hallein; Felix Stockenhuber, Oberpullendorf; Herbert Nell, Wien; Wolfgang Höller, Linz; Josef Würtz, Linz.

*Australia*

Abraham Rubinfeld, Royal Melbourne Hospital, Parkville; Christine Jenkins, Royal Prince Alfred Hospital, Camperdown; Peter Frith, Repatriation General Hospital, Daw Park; Anne Marie Southcott, Queen Elisabeth Hospital, Woodville; Philip Thompson, Sir Charles Gairdner Hospital, Nedlands; Charles Mitchell, Princess Alexandra Hospital, Woolloongabba; Mark Holmes, Royal Adelaide Hospital, Adelaide South Australia; Matthew Peters, Concord Repatriation General Hospital, Concord; Hamish Crawford, Liverpool Hospital, Liverpool; Peter Middleton, Westmead; Trevor Williams, The Alfred Hospital, Prahan; Mark Hurwitz, The Canberra Hospital, Woden.

*Canada*

Richard Hodder, Ottawa Civic Hospital, Ottawa, Ontario; Francois Maltais, Hospital Laval, Sainte-Foy, Quebec; Tharwat Fera, Vancouver, BC; Sheldon Mintz, Women's College Hospital, Toronto ON; Andrew McIvor, Queen Elizabeth II HSC, Halifax, NS; John Muscedere, Allergy & Respiratory Associates, Windsor, ON; David Small, SMBD Jewish General Hospital, Montreal PQ; Noe Zamel, Toronto Hospital Gen Division, Toronto, ON; Reza Maleki, Pulmonary Care Clinic Research Centre, Toronto ON; Emad Amer, Mississauga, ON; Robert Luton, London; Michael Alexander, Alexander Medical Innovations, Niagara Falls ON; Howard Conter, MSHJ Research Associates Inc., Halifax NS; Dan Dattani, Prairie Clinical Research Group C/O, Saskatoon SK; Gordon Ford, Rockyview General Hospital, Calgary AB; Jacques Hebert, Centre de Recherche, Ste-Foy PQ; Lawrence Homik, Concordia Hospital, Winnipeg MB; Fred Jardine, Summerhill Medical Clinic, Conception Bay NL; Darcy Marciniuk, Royal University Hospital, Saskatoon SK; Warren Ramesh, Links Clinic, Edmonton AB; Paolo Renzi, Centre de Recherche du CHUM, Montreal PQ; Brian Ramjattan, First Line Medical Services Inc., St. John's NL; Rizwan Somani, Glover Medical Clinic, Langley BC.

*France*

Emmanuel Weitzenblum, Hopital Hautepierre, Strasbourg; Christian Sevette, Centre Hospitalier, Perpignan; Pierre Zuck, Hôpital du Bon Secours, Metz; Henri Kafé, Cabinet de Pneumologie, Saint-Quentin; Bruno Housset, Hôpital Intercommunal, Créteil; Jacques Dupouy, Cabinet de Pneumologie, Nimes; Bernard Pigearias, Cabinet de Pneumologie, Nice; Jean-Michel Chavaillon, C.H. Antibes-Juan-les-Pins, Antibes; Jean-Paul Moreau, Cabinet de Pneumologie, Rennes; CH. Pison, C.H.R., La Tronche; Herve Jullian, Centre Hospitalier General, Martigues; Eric Fournier, Polyclinique de Henin Beaumont, Henin Beaumont; Dominique Lejay, Lejay Dominique, Vieux Conde; Laurent Fouquert, Cabinet de Pneumologie, Grasse; Lucien Bernabeu, Centre Hospitalier de Chauny, Chauny; Bernard Pigearias, Cabinet de Pneumologie, Nice; Jean-Marc Degreef, Centre Hospitalier Dr. Duchenne, Boulogne/Mer; Denis Vincent, Hopital Gaston Doumergue, Nimes; Jésus Gonzalez, Groupe Hospitalier, Paris; Nicolas Roche, Hotel dieu, Paris; Hervé Mal, Hopital Beaujon, Clichy; Christophe Verkindre, Centre Hospitalier de Béthune, Beuvry.

*Hungary*

Katalin Puha, Petz Aladár County Hospital, Györ; Teréz Kecskés, Pécs Institute of Pulmonology, Pécs; Ilona Vinkler, Szabolcs-Szatmár-Bereg County Jósa Andrá, Nyíregyháza; Zsuzsanna Mark, Pulmonology Hospital, Törökbálint; Katalin Major, Institut of Pulmonology, Budapest XI; Zsuzsanna Gönczi, Outpatient Dept. of Pulmonology Budapest, Budapest.

*Italy*

Adalberto Ciaccia, Arcispedale S. Anna, Ferrara; Gennaro D'Amato, A.O. Ospedale Cardarelli, Napoli; Giuseppe Di Maria, Ospedale Ascoli Tomaselli, Catania; Dario Olivieri, Ospedale Rasori, Parma; Leonardo Fabbri, Università di Modena e Reggio Emilia, Modena; Anna Maria Santolicandro, Azienda Ospedaliera "Spedali Civili", Livorno; Vincenzo Bellia, A.O. V. Cervello, Palermo; Enrico Clini, Villa Pineta Fondazione ONLUS, Gaiato, Marco Confalonieri, Ospedale Santorio, Trieste; Virginia De Rose, Ospedale S. Luigi, Orbassano; Franco Falcone, Ospedale Bellaria-Azienda USL, Citta di, Bologna; Fiorino Fiorentini, Ospedale "G.B. Morgagni", Forli; Vincenzo Fogliani, Az. USL 5 Presidio Ospedaliero, Milazzo; Giuseppe Idotta, Presidio Ospedaliero di Cittadella, Cittadella; Alessandro Galantino, A.O. Sandro Pertini, Roma; Anna Maria Moretti, Osp. S. Paolo, Bari; Massimo Pistolesi, Universitá degli Studi di Firenze, Firenze; Mario Polverino, ACISMOM, Cava De'Tirreni; Alfredo Potena, Arcispedale S. Anna, Ferrara; Ernesto Pozzi, Policlinico S. Matteo, Pavia; Giovanni Puglisi, A.O. "S. Camillo-Forlanini", Roma; Andrea Rossi, Ospedali Riuniti di Bergamo, Bergamo; Marcello Rossi, Policlinico delle Scotte-AO Senese, Siena; Giorgio Santelli, Ospedale Regionale "Ca' Foncello", Treviso; Matteo Scarpitta, P.O. da Procida, Salerno; Gianfranco Tassi, Spedali Civili, Brescia; Umberto Vincenzi, A.O. Ospedali Riuniti di Foggia, Foggia; Luigi Zucchi, Arcispedale S. Maria Nuova, Reggio Emilia; Renzo Zuin, Az. Ospedaliera di Padova, Padova; Marco Confalonieri, Ospedale Cattinara, Trieste.

*Ireland*

Stephen Lane, Adelaide & Meath Hospital, Dublin.

*Netherlands*

JPHM Creemers, Catharina Ziekenhuis, Eindhoven; CS de Graaff, Medisch Centrum Alkmaar, Alkmaar; Hans Hendrik, Timmer, Streekziekenhuis Midden-Twente, Hengelo; R Aalbers, Martini Ziekenhuis, Groningen; Adrianus P Sips, Wijkgezondheidscentrum Lunetten, DS Utrecht; JA van Noord, Atrium Medisch Centrum, Heerlen; WR Pieters, Elkerliek Ziekenhuis, HA Helmond; Th A Bantje, Ziekenhuis de Baronie, Breda; SJM Gans, Gans, DG Harderwijk; D Cheung, Praxis Cheung, HA Capelle A/D Ijssel; RALM Stallaert, Westfries Gasthuis, lok Streek, NP Hoorn; DRAJ De Munck, Maxima Medisch Centrum, Veldhoven; Adolf Maria Gerardus, Hendriks, Ziekenhuis St. Jansdal, Harderwijk; D Cheung, Vlietland Ziekenhuis, BA Schiedam.

*Poland*

Anna Bochenek, Centrum Leczenia Chorob Cywilizacyjnych, Warsaw; Marek Piepiorka, Szpital Specjalistyczny-Oddzial, Wejherowo; Ewa Gross-Tyrkin, General Practice "Non Nocere", Gdansk; Barbara Kaczmarek-Czeczotka, NZOZ-Polimedica, Lodz; Pawel Miekus, Szpital Miejski w Gdyni-Oddzial, Gdynia; Iwona Grzelewska-Rzymowska, Uniwerytet Medyczny w Lodzi Kilnika, Lodz; Dariusz Nowak, Osrodek Diagnostyki i Leczenia, Lodz; Malgorzata Czajkowska-Malinowska, Centrum POCHP i Niewydolnosci Oddechowej, Bydgoszcz; Andrzej Dyczek, Malopolskie Centrum Medyczne, Krakow; Ewa Trebas-Pietras, Wojewodzki Szpital im. Kardynala, Lublin.

*Portugal*

Joao Almeida, Hospital Sao Joao, Porto; Fernando Rodrigues, Hospital Fernando Fonseca, Amadora; Joao Cardoso, Hospital de Santa Marta, Lisboa.

*Russia*

Grigoriy Arutunov, Russian State Medical, Moscow; Mikhail Baluda, Moscow State Medical and Stomatol. Univ., Moscow; Yuliya Popova, City Clinical Hospital #13, Moscow; Aleksandr Sinopalnikov, Military Institute of Advanced Training, Moscow; Boris Bart, Russian State Medical University, Moscow; Aleksandr Chuchalin, City Clinical Hospital N57, Moscow; Yuriy Belousov, Russian State Medical University, Moscow; Aleksandr Solomatin, City Clinical Hospital N61, Moscow; Evgeniy, Shmelev, City Clinical Hospital N 11, Moscow; Sergey, Malanichev, Clinico-Diagnostical Complex #1, Moscow.

*Spain*

F. Sánchez Toril, Hospital Arnau de Vilanova, Valencia; P. de Lucas, Hospital Gregorio Maranón, Madrid; Juan Bautista Galdiz, Hospital de Cruces, Baracaldo; Héctor Verea Hernandez-Hector, Hospital Juan Canalejo, La Coruna; Conrado Shum, Hospital General Universitario de Elche, Elche, Alicante; Antoni Torres, Hospital Clinic i Provincial, Barcelona; José Morera, Hospital Germans Trias i Pujol, Badalona, Barcelona; J.L. Carretero, Hospital del Rio Hortega, Valladolid; Jose Ramon Rodriguez Suarez, Hosp. Clínico Universitario de Santiago, Santiago de Compostela; Eleuterio Llorca, Hospital General de Elda, Elda; Santamaria Abad, Hospital Severo Ochoa, Leganes, Madrid; Jose Echave Sustaeta, Hospital Doce de Octubre, Madrid; Fernando Fuentes, Hospital Universitario Infanta Cristina, Badajoz; B. Steen, Fundación Hospital Alcorcón, Alcorcón, Madrid; P. Cabrera, Hospital de Gran Canaria Dr. Negrín, Las Palmas de Gran Canaria, Las Palmas; Pilar Berlinches, Hosp. Naval del Mediterráneo, Cartagena, Murcia; J.M. Marín-Trigo, Hosp. Univ. Miguel Servet., Zaragoza; Luis Valdes, Hospital de Conxo, Santiago de Compostela, La Coruna; A. Armedillo, Hosp. Universitario Puerta del Mar, Cádiz; E. Gutiérrez, Hosp. Virgen de la Concha, Zamora.

*South Africa*

E M Irusen, Tygerberg Hospital, Tygerberg, Cape Town; M Plit, Milpark Hospital, Parktown West, Johannesburg; Eric Bateman, UCT Lung Institute, Mowbray, Cape Town; Michiel Prins, FARMOVS PAREXEL, Bloemfontein; Analene Nel, Medinel Clinical Trial Center, Paarl, Cape Town; James Joubert, Tiervlei Trial Centre, Bellville, Cape Town; Ismail Aboobake, Abdullah, Saint Augustine Medical Centre 2, Berea, Durban; Mohamed Sabeer Abdool-Gaffar, Kingsway Clinical Trial Centre, Durban, Amanzimtoti; Sanet Visser, Pretoria; Gideon Eduard Naude, Unitas Hospital, Centurion, Pretoria; Michelle V Middle, South African Trial Centre, George; Gerhard Ras, Muelmed Medical Centre, C/O Netcare & Garsfontein Roads; Ismail Abdullah, Gatesville Medical Centre, Cape Town.

*Switzerland*

Edelbert Imhof, City Hospital Triemli, Zürich; Michael Tamm, Kantonsspital, Basel; Hans Ulrich Bettschen, Praxis Dr. Bettschen, Spiez; Jürg Barandun, Lungenzentrum Hirslanden, Zürich; Martin Anton Häcki, Facharzt FMH, Zürich; Urs Honegger, Practice Dr. Honegger, Zürich; Jean-Paul Ketterer, Pneumologue FMH, La Chauxe-de-Fonds; Werner Bauer, Lindenhofspital Bern, Bern; Âlbrecht Breitenbuecher, Kantonsspital, Bruderholz.

*United Kingdom*

PB Anderson, Northern General Hospital, Sheffield; Jonathan Hamling, Oldfield Surgery, Bath, Avon; David Dutchman, Roebuck House Surgery, Hastings; Narendra Savani, North London Clinical Studies Centre, Middlesex; Carol McKinnon, Castemilk Health Centre, Glasgow; Alun George, The Stanpole Medical Centre, Soham, Cambridgeshire; S.J. Langley, Wythenshawe Hospital, Manchester; G.B. Ambepitiya, St. Margaret's Hospital, Epping; Peter Calverley, University Hospital Aintree, Liverpool; Stuart Elborn, Belfast City Hospital, Belfast; Gerald Gibson, Freeman Hospital, Sir Willian Leech Cent, Newcastle-upon-Tyne; G.P. Mc Bride, International Centre for Life, Newcastel-upon-Tyne; John Langan, Bailleston Health Centre, Bailleston, Glasgow; Ann Millar, Southmead Hospital, Bristol; Chris Stenton, Out Patient Department, Newcastle-upon-Tyne.
